# Comparison of deep learning architectures for predicting amyloid positivity in Alzheimer’s disease, mild cognitive impairment, and healthy aging, from T1-weighted brain structural MRI

**DOI:** 10.3389/fnins.2024.1387196

**Published:** 2024-07-02

**Authors:** Tamoghna Chattopadhyay, Saket S. Ozarkar, Ketaki Buwa, Neha Ann Joshy, Dheeraj Komandur, Jayati Naik, Sophia I. Thomopoulos, Greg Ver Steeg, Jose Luis Ambite, Paul M. Thompson

**Affiliations:** ^1^Imaging Genetics Center, Mark and Mary Stevens Neuroimaging and Informatics Institute, Keck School of Medicine, University of Southern California, Marina del Rey, CA, United States; ^2^University of California, Riverside, CA, United States; ^3^Information Sciences Institute, University of Southern California, Marina del Rey, CA, United States

**Keywords:** Alzheimer’s disease, amyloid, 3D convolutional neural networks, deep learning, transfer learning, vision transformers

## Abstract

Abnormal β-amyloid (Aβ) accumulation in the brain is an early indicator of Alzheimer’s disease (AD) and is typically assessed through invasive procedures such as PET (positron emission tomography) or CSF (cerebrospinal fluid) assays. As new anti-Alzheimer’s treatments can now successfully target amyloid pathology, there is a growing interest in predicting Aβ positivity (Aβ+) from less invasive, more widely available types of brain scans, such as T1-weighted (T1w) MRI. Here we compare multiple approaches to infer Aβ + from standard anatomical MRI: (1) classical machine learning algorithms, including logistic regression, XGBoost, and shallow artificial neural networks, (2) deep learning models based on 2D and 3D convolutional neural networks (CNNs), (3) a hybrid ANN-CNN, combining the strengths of shallow and deep neural networks, (4) transfer learning models based on CNNs, and (5) 3D Vision Transformers. All models were trained on paired MRI/PET data from 1,847 elderly participants (mean age: 75.1 yrs. ± 7.6SD; 863 females/984 males; 661 healthy controls, 889 with mild cognitive impairment (MCI), and 297 with Dementia), scanned as part of the Alzheimer’s Disease Neuroimaging Initiative. We evaluated each model’s balanced accuracy and F1 scores. While further tests on more diverse data are warranted, deep learning models trained on standard MRI showed promise for estimating Aβ + status, at least in people with MCI. This may offer a potential screening option before resorting to more invasive procedures.

## 1 Introduction

According to the World Health Organization (2022), approximately 55 million individuals are now affected by dementia—a number expected to rise to 78 million by the year 2030. Alzheimer’s disease (AD)—the most prevalent type of dementia - accounts for around 60–70% of the overall number of cases (World Health Organization, 2022). The underlying cause of AD is linked to the abnormal accumulation of specific proteins in the brain, including beta-amyloid plaques (Jack et al., 2018). These plaques are insoluble and toxic to brain cells (Masters and Selkoe, 2012). Additionally, abnormal tau proteins aggregate within neurons, in the form of neurofibrillary tangles, disrupting molecular transport within cells (Johnson and Hartigan, 1999). To visualize the distribution of Aβ in the brain, positron emission tomography (PET) has been used, but radioactive tracers that are sensitive to amyloid and tau proteins must be injected into the bloodstream, and this is invasive. Amyloid-sensitive PET can map the spatial distribution of Aβ in the brain, revealing the extent of AD pathology. As amyloid, tau, and neurodegeneration (A/T/N) are all considered to be the defining biological characteristics of AD, a recent NIA-AA task force recommended (Jack et al., 2018; Revised Again: Alzheimer’s Diagnostic Criteria Get Another Makeover | ALZFORUM, 2023) that future AD research studies should measure these processes.

In line with *post mortem* maps of pathology, PET scans show a distinctive trajectory of pathology in AD, usually starting in the entorhinal cortex, hippocampus, and medial temporal lobes, and then spreading throughout the brain as the disease advances. Early neuropathological work by Braak and colleagues pieced together the typical progression patterns for amyloid and tau in the brain (leading to the so-called ‘Braak staging’ system; Braak and Braak, 1991; Braak and Braak, 1997; Braak, 2000; Thompson et al., 2004; Braak et al., 2006). This progression is associated with gradual clinical and cognitive decline. Although amyloid levels can be measured in living individuals using PET imaging with amyloid-sensitive ligands such as Pittsburgh compound B (PiB; Klunk et al., 2004) or florbetapir (Clark et al., 2011), amyloid-PET is expensive, not widely available, and involves an invasive procedure, as it requires the injection of radioactive compounds into the participant. Ground truth measures can be obtained by directly measuring amyloid levels in the cerebrospinal fluid (CSF) through a spinal tap or lumbar puncture. The efficiency of Aβ protein aggregate clearance can be assessed in cerebrospinal fluid (CSF; Tarasoff-Conway et al., 2015). CSF peptides, such as Aβ1-42, and hyperphosphorylated tau show correlations with amyloid plaques and neuronal tangles observed in brain autopsies (Nelson et al., 2007). These biomarkers are linked to cognitive decline, providing insights for early detection of AD. Despite providing accurate information, these procedures are highly invasive. Thus, there is a significant interest in developing a less invasive test for abnormal amyloid to screen individuals before resorting to more invasive testing methods. Standard anatomical MRI cannot directly detect amyloid, but the accumulation of Aβ leads to widespread brain cell loss, which manifests as atrophy on T1-weighted (T1w) MRI. This process is evident through the expansion of the ventricles and widening of the cortical sulci, and the pattern of Aβ accumulation closely matches the trajectory of cortical gray matter loss detectable on brain MRI (Thompson, 2007). As such, MRI markers may offer a potential avenue for less invasive screening of abnormal amyloid levels in individuals.

In Petrone and Casamitjana (2019), Petrone et al. conducted a study where they used neuroimaging to predict amyloid positivity in cerebrospinal fluid (CSF), using an established cutoff of >192 pg./mL. They studied 403 elderly participants scanned with MRI and PET. Brain tissue loss rates were longitudinally mapped using the SPM12 (SPM12 software - Statistical Parametric Mapping, 2014) software. A machine learning classifier was then applied to the Jacobian determinant maps, representing local rates of atrophy, to predict amyloid levels in cognitively unimpaired individuals. The longitudinal voxel-based classifier demonstrated a promising Area Under the Curve (AUC) of 0.87 (95% CI, 0.72–0.97). Even so, this prediction required longitudinal scans from the same individual, and was not applicable when a patient had only a baseline scan. The brain regions with the greatest discriminative power included the temporal lobes, basal forebrain, and lateral ventricles. In Pan et al. (2018), Pan et al. developed a cycle-consistent generative adversarial network (Cycle-GAN) to generate synthetic 3D PET images from brain MRI (i.e., cross-modal image synthesis). Cycle-GANs build on the GAN concept introduced by Goodfellow et al. (2014) and perform a form of ‘neural style transfer’ by learning the statistical relationship between two imaging modalities. In related work (Jin et al., 2023), we developed a multimodal contrastive GAN to synthesize amyloid PET scans from T1w MRI and FLAIR scans. For more details on image-to-image translation and the underlying mathematics, readers are referred to Qu et al. (2021) and Wang et al. (2020). Cross-modal synthesis is an innovative use of deep learning to generate synthetic PET images, offering potential applications in cases where PET scans may be challenging or costly to obtain.

In Shan et al. (2021), Shan et al. used Monte Carlo simulations with *k*-fold cross validation to predict Aβ positivity using domain scores from cognitive tests, obtaining an accuracy of 0.90 and 0.86 on men and women, respectively, with subjective memory complaints. In Ezzati et al. (2020), Ezzati et al. used an ensemble linear discriminant model to predict Aβ positivity using demographic information, ApoE4 genotype (as this is the major risk gene for late onset AD), MRI volumetrics and CSF biomarkers, yielding AUCs between 0.89 and 0.92 in participants with amnestic mild cognitive impairment (aMCI). In Kim S, et al. (2021), Kim et al. used a 2.5-D CNN (a convolutional neural network that operates on a set of 2D slices from a 3D volume) to predict Aβ positivity from [^18^F]-fluorodeoxyglucose (FDG) PET scans, with an accuracy of 0.75 and an AUC of 0.86. In Son et al. (2020), Son et al. used 2D CNNs to classify Aβ-PET images. They showed that in cases where scans present visual ambiguity, deep learning algorithms correlated better with ground truth measures than visual assessments. This underscores the potential of such algorithms for clinical diagnosis and prognostic assessment, particularly in scenarios where visual interpretation is challenging or uncertain. In Bae et al. (2023), Bae et al. used a deep learning based classification system (DLCS) to classify Aβ-positive AD patients vs. Aβ-negative controls using T1w brain MRI. and reported an AUC of 0.937. In Yasuno et al. (2017), Yasuno et al. conducted a correlation analysis between the T1w/T2w ratio and PiB-BP_ND_ values and found a significant positive relationship between the regional T1w/T2w ratio and Aβ accumulation. Their study concluded that the T1w/T2w ratio is a prospective, stable biological marker of early Aβ accumulation in cognitively normal individuals.

In our current study, we aimed to assess the effectiveness of a diverse range of deep learning architectures for predicting Aβ + from 3D T1w structural MRI. 3D convolutional neural networks (CNNs) have demonstrated success in detecting Alzheimer’s disease and in ‘brain age’ estimation from brain MRI (Lam and Zhu, 2020; Lu et al., 2022). CNNs learn predictive features directly from raw images, eliminating the need for extensive pre-processing, or visual interpretation of images. As Aβ + is weakly associated with age and regional morphometric measures (such as the volume of the entorhinal cortex), we incorporated these features as predictors as well. To achieve this, we compared the performance of classical machine learning algorithms—logistic regression, XGBoost, and shallow artificial neural networks—for the amyloid prediction task. We also evaluated a hybrid network that combines a CNN with a shallow artificial neural network. This merges numeric features, often called ‘tabular data’, with entire images, weighting each input type in proportion to its added value for the prediction task.

In our tests, we separately report accuracy for Aβ + prediction in healthy people vs. those who already show signs of clinical impairment (MCI and AD), as Aβ + prediction may be more challenging in controls. The now-standard biomarker model by Jack et al. (2018) posits that amyloid levels may begin to rise before neurodegeneration is apparent on MRI, although some researchers have challenged this sequence of events, noting that it may not be universal (Cho et al., 2024), especially in populations of non-European ancestry.

As deep learning models are often enhanced by “pre-training” (first training networks on related tasks), we evaluated the performance of the models when pre-training them to predict age and sex, using data from 19,839 subjects from the UK Biobank dataset (Sudlow et al., 2015). Transfer learning - an artificial intelligence/deep learning approach—has previously been shown to enhance MRI-based Alzheimer’s disease (AD) classification performance (Lu et al., 2022; Dhinagar and Thomopoulos, 2023). In transfer learning, network weights are first optimized on previous tasks and then some network layers have their weights ‘frozen’—held constant—while others are adjusted when training the network on the new task. There is a debate about when such pre-training techniques enhance performance on downstream tasks, especially when the tasks differ. Our study aimed to investigate whether these pre-training techniques help in predicting amyloid positivity. We examined whether the amount of data used for the pretraining task impacts the accuracy of the downstream task after fine-tuning. This evaluation assessed transfer learning for predicting Aβ + from structural MRI.

Finally, Vision Transformers (ViTs) have shown enormous success in computer vision, and more recently in medical imaging (Matsoukas, 2021). Unlike CNNs, ViTs employ a self-attention mechanism to capture long-range spatial dependencies in an image, providing a more comprehensive global perspective (Li, 2022). This property can help in medical imaging tasks, where anatomical context and spatial patterns can be crucial. Even so, effective training of ViTs typically requires a very large number of MRI scans (Bi, 2022; Jang and Hwang, 2022; Willemink et al., 2022). In Dhinagar et al. (2023), the ViT architecture was used to classify AD vs. healthy aging, achieving an AUC of 0.89. Building on this, our investigation aimed to assess the performance of the ViT architecture in predicting Aβ + from T1w MRI. We conducted a benchmark comparison with the commonly used CNNs, to compare these two architectures for Aβ + prediction.

With the advent of new anti-Alzheimer’s treatments effectively targeting amyloid pathology, there is increasing interest in predicting Aβ + using less invasive and more accessible brain imaging techniques, such as T1-weighted MRI. In this work, we compare multiple machine learning and deep learning architectures, including, (1) classical machine learning algorithms, such as logistic regression, XGBoost, and shallow artificial neural networks, (2) deep learning models based on 2D and 3D convolutional neural networks (CNNs), (3) a hybrid ANN-CNN, combining the strengths of shallow and deep neural networks, (4) transfer learning models based on CNNs, and (5) 3D Vision Transformers, to infer Aβ status from standard anatomical MRI. We hypothesize that methods (1), (3) and (5) will perform best.

## 2 Imaging data and preprocessing steps

The Alzheimer’s Disease Neuroimaging Initiative (ADNI) is a comprehensive, multisite study initiated in 2004, at 58 locations across North America. It aims to collect and analyze neuroimaging, clinical, and genetic data to identify and better understand biomarkers associated with healthy aging and AD (Veitch et al., 2019). In our analysis, we examined data from 1,847 ADNI participants with a mean age of 74.04 ± 7.40 years (863 females and 984 males). We included participants from all phases of ADNI (1, 2, GO and 3) who had both MRI and PET scans. The data was acquired across 58 sites with (both 1.5 and 3 T) GE, Siemens or Philips scanners. Forty of these sites had a change in scanner manufacturer or model across the scanning time of our subset. The distribution of participants included 661 cognitively normal (CN) individuals, 889 with mild cognitive impairment (MCI), and 297 with dementia. Overall, the dataset included 954 individuals classified as Aβ + (amyloid positive) and 893 as Aβ- (amyloid negative). A detailed table with the subject demographic breakdown can be found in Table 1.

**Table 1:** Demographic data of individual train, validation and test set.

Individual distribution	Total N	Sex		Mean age ± St. Dev.	Amyloid classification		Diagnosis		
		M	F		+ve	-ve	CN	MCI	Dem
Train	1,292	680	612	73.99 ± 7.43	662	630	465	630	197
Validation	278	154	124	74.12 ± 6.95	146	132	105	126	47
Test	277	150	127	74.20 ± 7.74	146	131	91	133	53

In ADNI1, participants initially underwent PiB scans instead of florbetapir scans (ADNI, n.d.). However, the protocol was amended before the study’s conclusion to transition to florbetapir scans due to processing time constraints. Consequently, PiB scans were only collected from ADNI1 participants. For participants in ADNI1 who transitioned into ADNIGO and then ADNI2, initial PET scans occurred 2 years from the date of the last successful florbetapir and FDG-PET scan conducted under ADNIGO. Additionally, in ADNI1, only a subset of participants received FDG scans. In ADNI2, subjects underwent up to 3 florbetapir scans and up to 2 FDG scans, with each scan acquired at 2-year intervals. These scans were conducted within a two-week window before or after the in-clinic assessments at Baseline and at 24 months after Baseline. In ADNI3, both Tau and Amyloid imaging were conducted on all participants during their initial ADNI3 visit. Amyloid PET imaging was carried out every 2 years using florbetapir for participants continuing from ADNI2 or florbetaben for newly enrolled participants (ADNI, n.d.). ADNI does not perform partial volume correction for amyloid PET analysis. It also does not account for off-target binding.

Mild cognitive impairment (MCI) is an intermediate state between normal aging and AD (Petersen et al., 1999), and is a significant focus in clinical trials, as many trials enroll individuals with MCI as they are assumed to be more likely to respond to therapy than people already diagnosed with AD. In the construction of the final dataset, we excluded participants who lacked basic clinical information or had poor-quality imaging data, such as scans with severe motion, distortion, or ringing artifacts.

ADNI has more participants with MCI compared to those with AD or CN. This is partly due to the initiative’s focus on the early stages of cognitive decline and the progression to Alzheimer’s disease. From ADNI phase 1 onward, twice as many MCI subjects were enrolled than AD cases or controls, with a target enrolment ratio of 1:2:1 for controls:MCI:AD. This higher proportion of MCI participants aligns with ADNI’s objective to study factors that influence disease progression from MCI to AD, which is critical for early diagnosis and intervention.

Having a balanced number of participants in each diagnostic class and repeating the experiments could in principle lead to more reliable and generalizable models, reducing the bias toward the more prevalent class, MCI. But balancing the datasets can come with its own set of challenges. One issue might be the reduced amount of training data if undersampling is used to balance the classes, which can lead to loss of information, especially as the dataset is not large to begin with. Alternatively, oversampling/differential sampling methods such as SMOTE, or generative models such latent diffusion models, denoising diffusion probabilistic models (DDPMs), or VAEs might be used to generate synthetic data for the underrepresented classes, to augment the training set, but this might also introduce noise and overfitting.

T1w MRI scans were further processed using the automated segmentation software package FreeSurfer (Fisch, 2012), following the ENIGMA standardized protocol for brain segmentation and quality assurance (Van Erp and Hibar, 2016; van Erp et al., 2018).^1^ The segmentations of subcortical regions (including lateralized hippocampus) and cortical regions [based on the Desikan-Killiany (DK) atlas regions (Desikan et al., 2006); including entorhinal cortex] were extracted and visually inspected for accuracy. The CSF, white and gray matter segmentations were extracted and visually inspected for each subject using FSL’s Fast function.^2^

**Figure 1:**
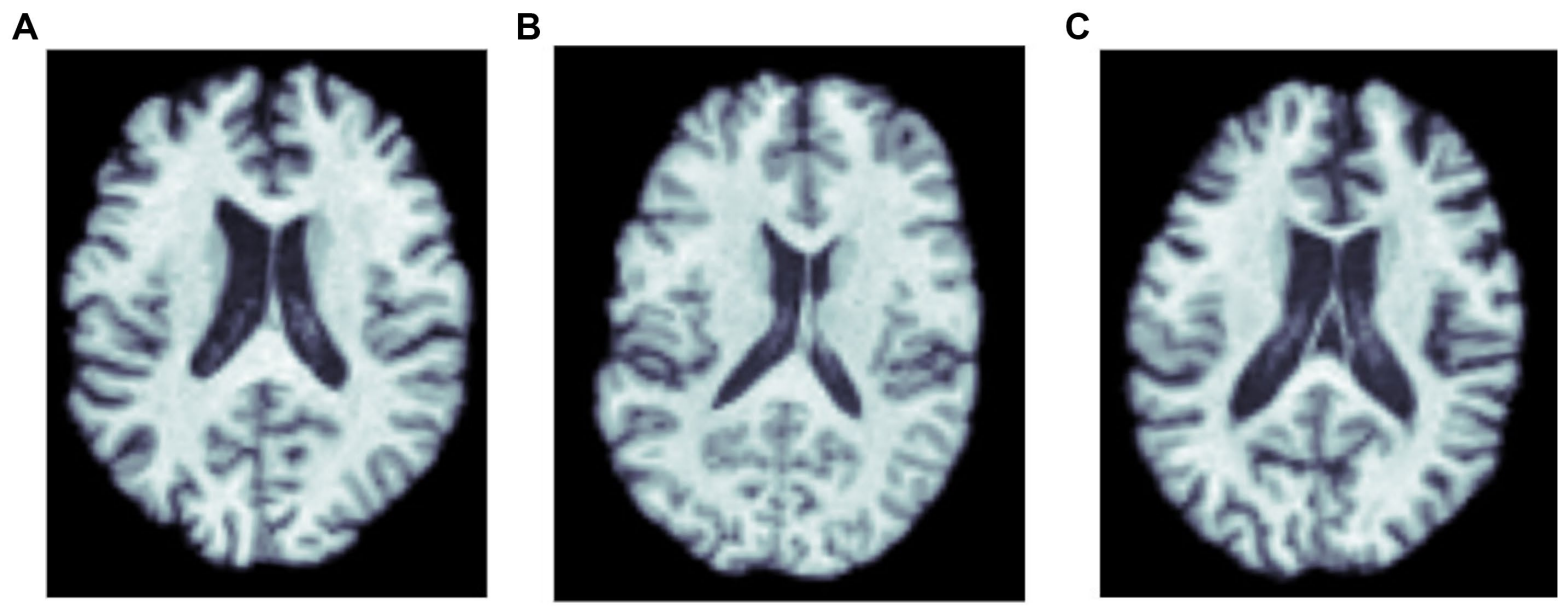
MRI scans of three amyloid positive participants: **(A)** a cognitively normal control, and participants diagnosed with **(B)** MCI, and **(C)** dementia.

For training the CNN architectures, we used part of this dataset, so that an independent subset of the data could be reserved for testing. We focused on 3D T1w brain MRI scans (see Figure 1) from 762 subjects, with a mean age of 75.1 ± 7.6 years (394 females, 368 males). This subset included 459 cognitively normal controls, 67 individuals with MCI, and 236 with AD. These participants were selected as they also had amyloid-sensitive PET scans collected close to the time of the T1w MRI acquisition, with a maximum interval between scans set to 180 days (We note that one could consider an extension of the current problem, where the interval from the MRI to the amyloid assessment is considered as a variable, *t*, and used as input in the model, where *t* may be positive or negative). No repeated scans were used for the CNNs. The restriction on the time interval between scans was intended to help in estimating the relation between MRI features and amyloid positivity. As ViTs are more data intensive architectures, the whole dataset - with repeated scans - was used to train them. The test dataset in that case was designed to not have repeated scans, or scans from subjects in training or validation sets. Thus, the training dataset had 1,290 T1w MRI scans from 845 individual subjects, the validation dataset had 276 T1w MRI scans, and the test dataset had 275 T1w MRI scans. For the transfer learning experiments, we used data from 19,839 subjects from the UK Biobank dataset (age: 64.6 ± 7.6 years) comprising 10,294 females and 9,545 males.

As is customary when benchmarking deep learning methods, the 3D T1w brain MRI scans underwent a series of pre-processing steps (Lam and Zhu, 2020). These steps included nonparametric intensity normalization using N4 bias field correction, ‘skull stripping’ for brain extraction, registration to a template using 6 degrees of freedom (rigid-body) registration, and isometric voxel resampling to 2 mm. The resulting pre-processed images were of size 91x109x91. Furthermore, the T1w images underwent min-max scaling so that all values ranged between 0 and 1. This normalization process is common in image processing (and is similar to batch or instance normalization in deep learning), allowing standardized and consistent representation of image intensity values, which may aid in subsequent analyses and model training. The preprocessing pipeline applied to the 3D T1w MRI images ensures that the background of the scans is 0 intensity, and due to the normalization of input before CNN model, ideally, the effect of the original background or intensity range of the scan on performance of convolution models is negligible. To ensure a direct correspondence with the patch sizes used for the ViT models, the T1w input scans were resized to dimensions of both 64x64x64 and 128x128x128 for the ViT experiments. This resizing ensures compatibility between the image dimensions and the patch sizes employed in the ViT models, and allowed us to consistently integrate the T1w images into the analysis pipeline.

As is the convention in the ADNI dataset, two cut-off values were employed, providing alternative definitions of amyloid positivity, based on PET cortical *standardized uptake value ratio* (SUVR; denoted Αβ_1 by ADNI). For the 18F-florbetapir tracer, amyloid positivity was determined using mean 18F-florbetapir, with Aβ + defined as >1.11 for cutoff_1 and > 0.79 for cutoff_2. When florbetaben was used, Aβ + was defined as >1.20 for cutoff_1 and > 1.33 for cutoff_2. The SUVR values were normalized by using a whole cerebellum reference region (Hansson et al., 2018; Blennow et al., 2019). Each of these two cutoffs has been employed in the literature to define amyloid positivity, and to establish eligibility criteria for anti-amyloid drug treatments (van Dyck et al., 2023).

## 3 Models and experiments

### 3.1 Classical machine learning algorithms

As the first set of methods to evaluate for predicting Aβ + from anatomical MRI, we employed the following three classical machine learning algorithms: logistic regression, XGBoost, and a fully-connected artificial neural network (ANN) with 7 hidden layers. The ANN incorporated a Rectified Linear Unit (ReLU) activation function between layers. As predictors, we used measures that have previously been associated with amyloid levels in the literature: age, sex, clinical diagnosis, ApoE4 genotype values (2 for two copies of the ApoE4 allele and 1 for one E4 allele, 0 otherwise), overall volumes of cerebrospinal fluid (CSF), gray and white matter (all estimated from the brain MRI scan), as well as the left and right hippocampal and entorhinal cortex volumes. Regional volumes were extracted from the T1w MRI using FreeSurfer and were available for the entire brain. Previous studies like Kai et al. (Hu et al., 2019) and Thompson et al. (2004) show that hippocampal and entorhinal cortex volumes are among the most consistently affected in Alzheimer’s disease, and as a result we focused on those two regional volumes in our study. The dataset was partitioned into independent training, validation, and testing sets, approximately in the ratio of 70:20:10. Standard performance metrics for the three algorithms (balanced accuracy and F1 Score on the test dataset), were computed to assess their effectiveness in predicting amyloid positivity.

### 3.2 2D CNN architecture

**Figure 2:**
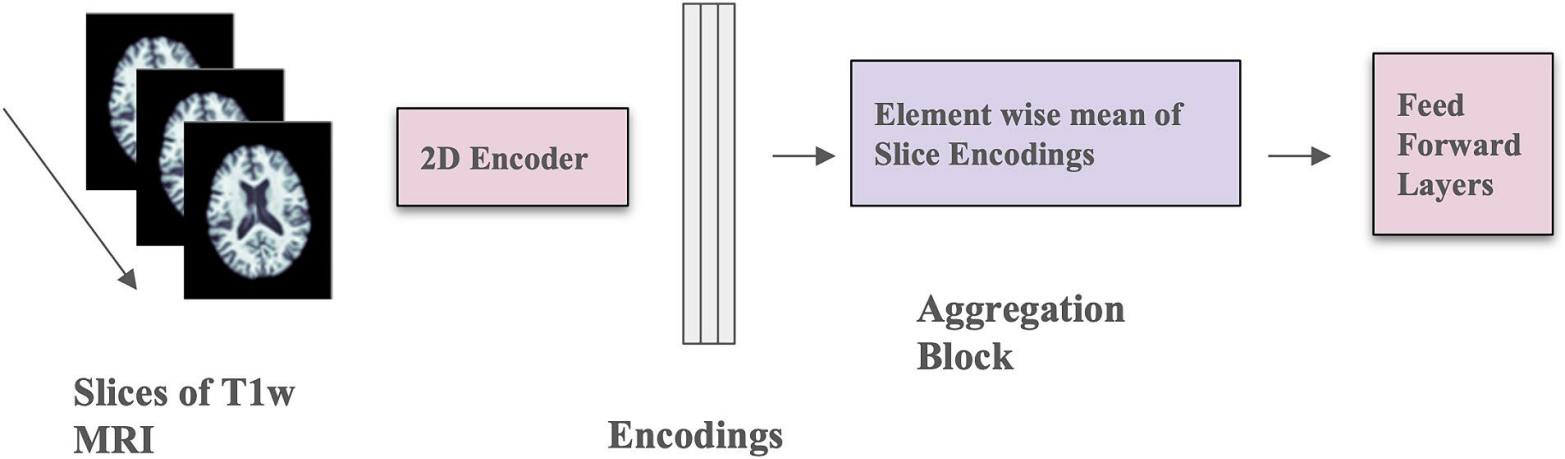
Model architecture with mean-based aggregation. The two pink blocks include trainable parameters; the purple block is a deterministic operation.

We implemented the 2D CNN architecture that we proposed in Gupta et al. (2021). In this model, 3D scans are used as the input, but each slice is encoded using a 2D CNN encoder (see Figure 2), which makes the training faster, requires less RAM, and allows pre-training using foundation models trained on large datasets of 2D photographic images, such as ImageNet. The encoded slices are then combined through an aggregation module that employs permutation-invariant layers, ultimately producing a single embedding for the entire scan. This embedding was then passed through feed-forward layers to predict whether the individual was amyloid positive or negative. This architecture allows for effective representation learning from 3D scans, and the aggregation module captures information from individual slices to predict amyloid status.

The 2D CNN encoder processes a single 2D slice as input and generates a *d*-dimensional embedding for each slice. The number of filters in the last layer of the architecture is *d*, determined by the dimension of the output from the aggregation module. The aggregation module incorporates permutation-invariant layers, ensuring that the output remains independent of the slice order. Specifically, the element-wise mean of all slice encodings is computed and used as the permutation-invariant layer. The value of *d* is fixed at 32, and a feed-forward layer with one hidden layer containing 64 activations is used. The slices in this context are sagittal. This model was trained for 100 epochs using the Adam optimizer (Kingma and Ba, 2015), a weight decay of 1×10^−4^, a learning rate of 1×10^−4^, and a batch size of 8. Mean squared error loss was employed as the optimization function during training. Model performance was measured using balanced accuracy.

### 3.3 3D CNN architecture

**Figure 3:**
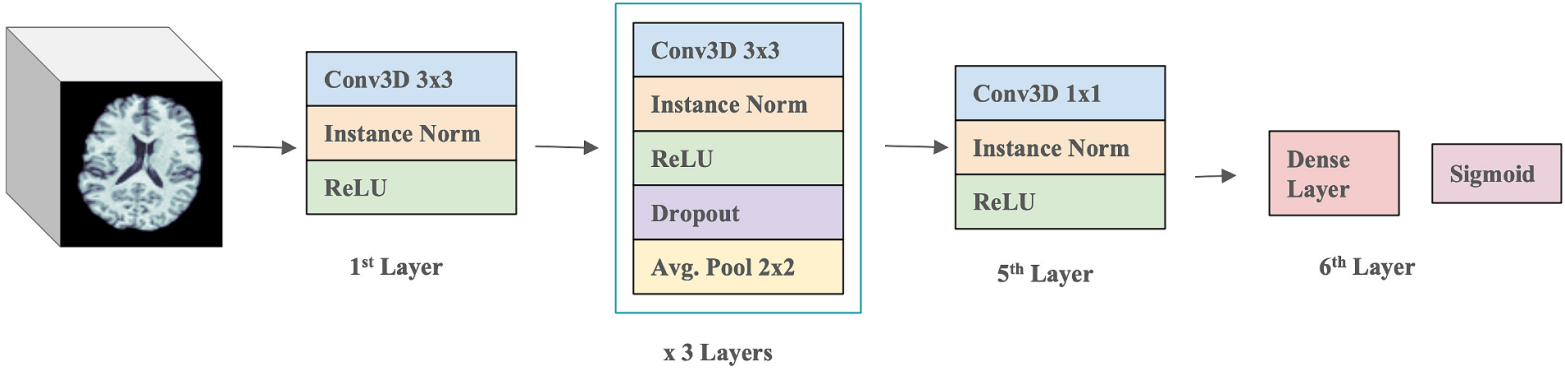
3D CNN model architecture.

The 3D CNN was composed of four 3D Convolution layers with a filter size of 3 × 3, followed by one 3D Convolution layer with a 1 × 1 filter, and a final Dense layer with a sigmoid activation function (see Figure 3). A ReLU activation function and Instance normalization were applied to all layers. Dropout layers (with a dropout rate of 0.5) and a 3D Average Pooling layer with a 2 × 2 filter size were introduced into the 2nd, 3rd, and 4th layers. During training, models were optimized with a learning rate of 1×10^−4^. Test performance was evaluated using balanced accuracy and F1 Score. To address overfitting, both L1 and L2 regularizers were employed, along with dropouts between layers and early stopping. Youden’s *J* index (Youden, 1950) was used to determine the threshold for binary classification of Aβ + during testing, allowing comparison with the true cutoff values. Hyperparameter tuning was conducted through *k*-fold cross-validation to optimize model performance.

### 3.4 Hybrid CNN architecture

**Figure 4:**
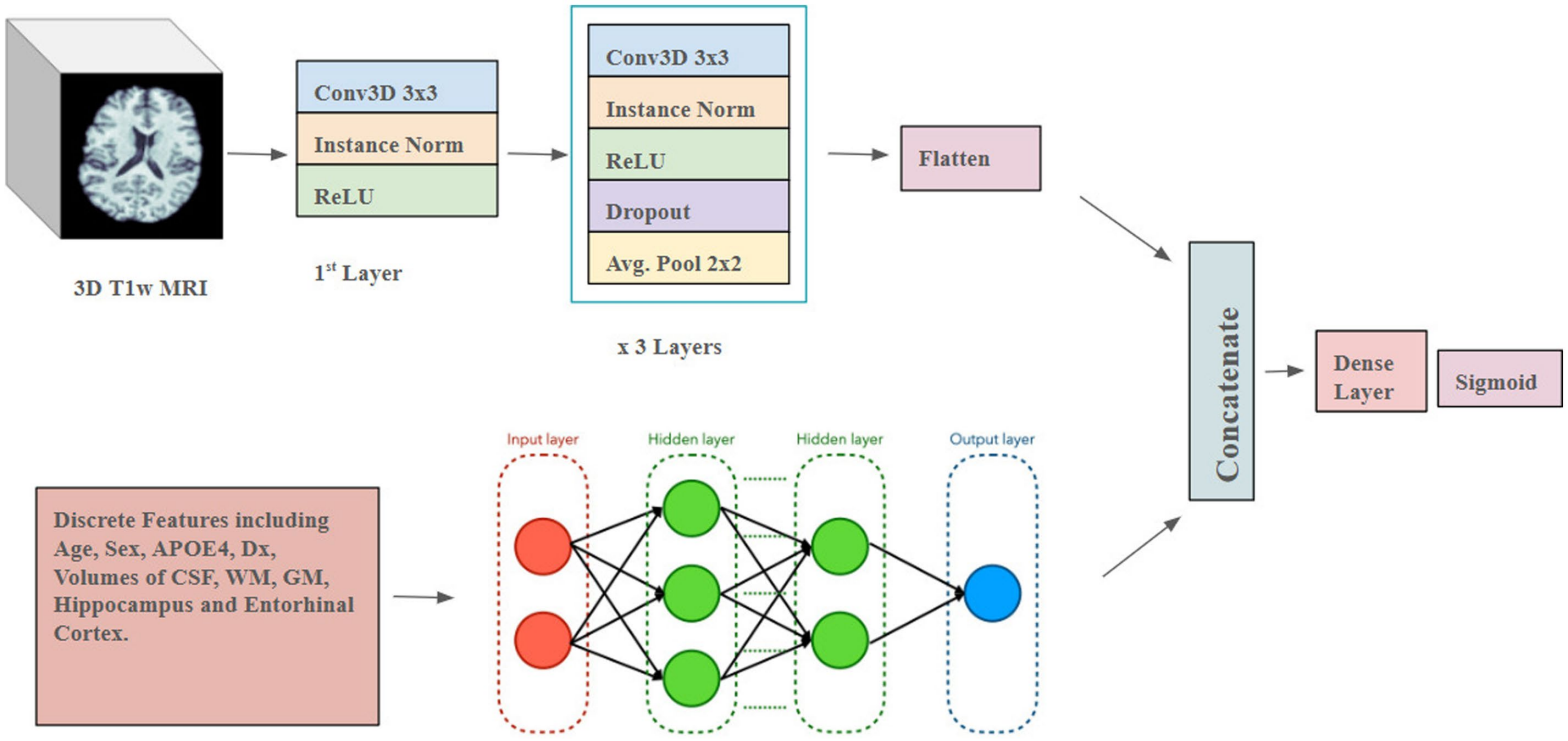
Hybrid 3D CNN model architecture.

The hybrid model (Figure 4) combines a 3D CNN using T1w images as input with an ANN that takes discrete, tabular data (which consists of simple values that are numeric or categorical) including age, sex, diagnosis, APOE4 values (2 for two copies of E4, 1 for one E4, and 0 for none), overall volumes of CSF, white and gray matter, and left and right hippocampal and entorhinal cortex volumes. The 3D images and the derived discrete data were fed into individual models, separately. After passing through flattening layers in the 3D CNN, the layers from the ANN are stacked with the tensors from the 3D CNN. Subsequently, the combined data passes through further Dense layers to predict Aβ+. The learning rate was set to 0.001, and the Adam Optimizer was used, with a batch size of 2. The model was trained for 200 epochs. The 3D CNN model consisted of 3 convolution blocks with increasing filter sizes (32, 64, 128, and 256) along with Batch Normalization and Max Pooling. The final convolution layer, before concatenation, had a filter size of 256 and used average pooling. The ANN had three layers with hidden layer sizes of 1,024, 512, and 64, along with instance normalization and the ReLU activation function.

This hybrid model was executed separately for both entorhinal cortex and hippocampus volumes, as well as in combination. In the combined case, we also considered the case where APOE genotype values were excluded from the discrete features input. Performance was evaluated using balanced accuracy and F1 Score, to compare the four models.

### 3.5 Vision transformers

**Figure 5:**
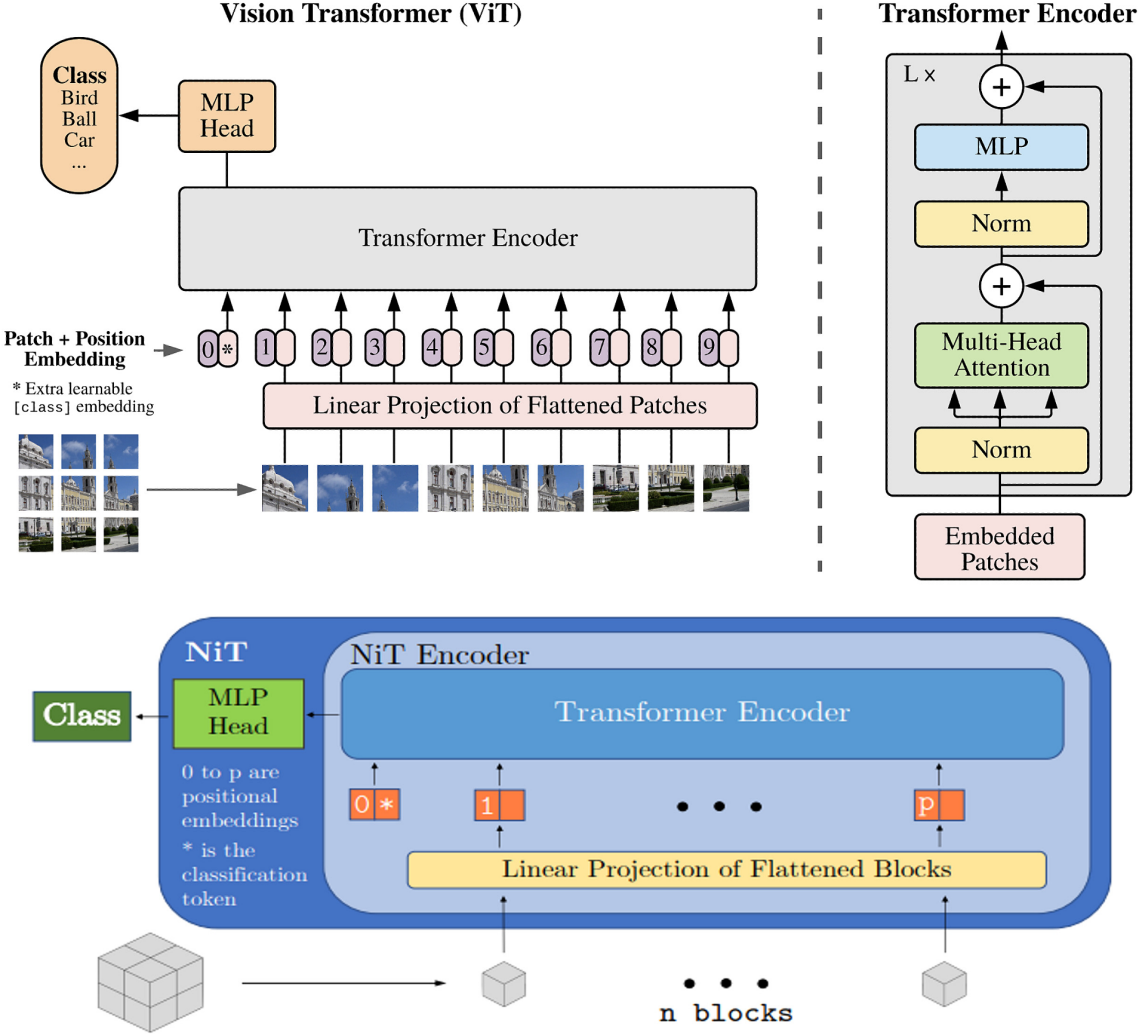
Overview of the vision transformer architecture, : reproduced from Singla et al. (2022).

We trained two variations of the ViT architecture: (i) the neuroimage transformer (NiT) and (ii) the multiple instance NiT (MINiT; Singla et al., 2022), as illustrated in Figure 5. These architectures involve several key steps. Initially, the input image is split into fixed-sized patch embeddings. These patches are then combined with learnable position embeddings and a class token. The resulting sequence of vectors is fed into a transformer encoder, consisting of alternating layers of multi-head attention and a multi-layer perceptron (MLP; *top right*, Figure 5). This architecture has been adapted to accommodate patches (cubes) from 3D scans. The NiT model was configured with a patch size of 8x8x8, without any overlap, a hidden dimension size of 256, six transformer encoder layers, and between 2 and 12 self-attention heads, with a dropout rate of 0.3.

Based on MiNiT (Singla et al., 2022), the input image, represented as M ∈ ℝ*^L × W × H^*, is transformed into a sequence of flattened blocks. If (*B,B,B*) denotes the shape of each block, the number of blocks is *LWH/B*^3^. Non-overlapping cubiform patches are extracted from the input volume and flattened. These patches are then projected to *D* dimensions, the inner dimension of the transformer layers, using a learned linear projection. The generated sequence of input patches is augmented with learned positional embeddings for positional information and a learned classification token. Subsequently, this sequence is fed into a transformer encoder comprising *L* transformer layers. Each layer consists of a multi-head self-attention block and a multi-layer perceptron (MLP) block, which incorporates two linear projections, with a Gaussian Error Gated Linear Unit (GEGLU) nonlinearity applied between them. Layer normalization is applied before - and residual connections are added after - every block in each transformer layer. Finally, a layer normalization and an MLP head consisting of a single *D × C* linear layer project the classification token to ℝ*^C^*, where *C* represents the number of classes (Singla et al., 2022).

The NiT architecture served as the primary model in our experiments, and we fine-tuned the default values for the number of transformer encoder layers and attention heads. In the case of MINiT, as well as incorporating a learned positional embedding on the training data to patches and adding a learned classification token to their sequence, a learned block embedding was also introduced (Singla et al., 2022). This embedding was included to retain the positional information of the block within the neuroimage of each patch. MINiT adopted similar parameters to those described for NiT.

We also performed hyperparameter selection for both models through a random search within specified upper and lower bounds. These parameters included the learning rate (chosen from a uniform distribution between 0.00001 to 0.001), weight decay (selected from a uniform distribution between 0.00001 to 0.001), the number of warm-up epochs (options included 1, 5, 16), the number of attention heads (options included 2, 4, 8, and 12), and the number of encoder layers (choices were 3, 4, and 6). These hyperparameters were defined based on the bounds typically used in ViT architectures (Bi, 2022; Jang and Hwang, 2022). We used the Adam optimizer (Kingma and Ba, 2015).

After training, we tested the model on the hold-out test dataset. We evaluated model performance with several metrics including the receiver-operator characteristic curve-area under the curve (ROC-AUC), accuracy, and F1-score. We determined the threshold for these metrics was accomplished through Youden’s Index (Youden, 1950).

## 4 Results

In the comparison of classical machine learning models for predicting amyloid positivity, the best results were achieved with the artificial neural network (ANN), yielding a balanced accuracy of 0.771 and an F1 score of 0.771. The balanced accuracy values for the classical models ranged from 0.69 to 0.77, indicating predominantly similar classification performances across these models (Table 2).

**Table 2:** Balanced accuracy (BA) and F1 scores for classical machine learning models.

	XGBoost	Logistic regression	ANN
	BA / F1 score	BA / F1 score	BA / F1 score
Data except for EC volume	0.742 / 0.678	0.770 / 0.734	0.711 / 0.696
Data except for HP volume	0.742 / 0.689	0.770 / 0.734	0.711 / 0.696
Data except for GM, WM and CSF volumes	0.697 / 0.656	0.770 / 0.734	0.771 / 0.771
Data with all features	0.756 / 0.701	0.770 / 0.734	0.725 / 0.716

The best performance was obtained with the ANN where all data except GM, WM and CSF volumes are considered, giving a balanced accuracy of 0.771.

The 2D CNN performed worse than the classical machine learning algorithms. Across an average of three runs, the model achieved a test accuracy of 0.543. In contrast, the 3D CNN architecture performed better, as indicated in Table 3. The Youden’s *J* Index, used to determine the threshold for classifying Aβ + as 0/1 based on MRI scans, varied across different subject groups. Specifically, it was found to be 0.605 when considering only MCI and AD participants, 0.509 for cognitively unimpaired controls (CN), and 0.494 when considering all subjects. A balanced accuracy score of 0.760 was achieved for classification when all subjects were included. The accuracy increased to 0.850 when classifying individuals with only MCI or AD. In the case of CN, the balanced accuracy was 0.631. This observation aligns with expectations, as classifying Aβ + is more challenging in the earlier stages of the disease. According to the now-accepted Jack et al. model of the sequence of biomarker elevation in AD (Jack et al., 2018), abnormal amyloid accumulation typically precedes extensive brain atrophy, although individuals may vary in the order and relative intensities of these processes.

**Table 3:** 3D CNN results for all subjects, and with CN and MCI/AD groups considered separately.

	All subjects	CN	MCI and AD
Balanced accuracy	0.760	0.631	0.850
F1 score	0.746	0.480	0.824

The hybrid model performed better than the 3D CNN model (Table 4). The hybrid model gave the best balanced accuracy of 0.815, when using hippocampal volume in the predictor set. Considering the CN, MCI and AD subjects in the test set separately for this model, the balanced accuracies are 0.616, 0.75 and 0.85 respectively, while the F1 Scores are 0.4, 0.969 and 0.863, respectively. This observation aligns with expectations, as classifying Aβ + is more challenging at the earlier stages of the disease.

**Table 4:** Balanced accuracy and F1 score for the hybrid model architecture.

	Entorhinal cortex volume	Hippocampus volume	Entorhinal cortex and hippocampus volume
Balanced accuracy	0.759	0.815	0.787
F1 score	0.746	0.793	0.769

The results comparing various hyperparameters for both NiT and MINiT model architectures are summarized in Table 5. Four different hyperparameter tunings were evaluated for both image sizes. In contrast, the NiT architecture performed more poorly, with classification accuracies close to chance (ranging between 0.5 to 0.6) across different hyperparameters and two image sizes. The MINiT architecture outperformed the NiT architectures, particularly for the image size of 64x64x64, with a test accuracy of 0.791 and a test ROC-AUC of 0.857. Therefore, the MINiT architecture improved upon the NiT architecture.

**Table 5:** Experimental results for NiT and MINiT models.

Arch.	Image size	Hyperparameters of transformer architectures				Test ROC-AUC	Test balanced accuracy	Test F1 score
		Transformer layers	Attention heads	Dimension	MLP dimension			
NiT	(64)^3^	512	3	12	175	0.494	0.541	0.614
		256	6	8	64	0.579	0.592	0.609
		256	4	8	234	0.485	0.516	0.221
	(128)^3^	512	3	12	175	0.569	0.581	0.600
		256	6	8	64	0.692	0.590	0.584
		256	4	8	234	0.692	0.468	0.495
MINiT	(64)^3^	6	12	256	309	**0.857**	**0.791**	**0.793**
		6	8	256	309	0.755	0.697	0.674
		6	8	128	128	0.585	0.599	0.686
		6	12	258	128	0.794	0.776	0.782
	(128)^3^	6	12	256	309	0.503	0.534	0.557
		6	8	256	309	0.668	0.649	0.688
		6	8	128	128	0.799	0.747	0.766
		6	12	258	128	0.476	0.527	0.584

Columns 3 to 6 show the hyperparameters of the transformer architectures, namely Transformer Layers, No. of Attention Heads, Dimension and MLP Dimension. The experiments are compared using test ROC-AUC, accuracy and F1 Score. Bold numbers indicate the best results.

Hyperparameter tuning of attention heads, learning rate, encoder layer, and weight decay all enhanced model performance. Notably, the performance for the downscaled image of size 64x64x64 was superior to that for the upsampled image of size 128x128x128, in our experiments.

## 5 Discussion

This work, and several more recent amyloid-PET studies, show that the pattern of Aβ accumulation closely matches the anatomical trajectory of cortical gray matter loss detectable on brain MRI, a process that is also evident through the widening of the cortical sulci over time. Although the now widely accepted biomarker model by Jack et al. (2013) suggests that amyloid levels become statistically abnormal earlier than MRI measures of atrophy, all the processes occurring, to some extent, simultaneously. The order in which we detect them with imaging also depends, to some extent, on the sensitivity of our measurement techniques. Magnetic resonance imaging (MRI) measures of atrophy may not be as sensitive as amyloid positron emission tomography (PET) in detecting early changes, as amyloid levels typically become statistically abnormal earlier than structural atrophy becomes abnormal on MRI. The sensitivity of the imaging modality used plays a role in determining the order in which the pathological changes are observed, in addition to the temporal ordering of the underlying biological processes. There have been successful attempts to predict amyloid positivity in patients with MCI using radiomics and structural MRI (Petrone and Casamitjana, 2019; Kim J P, et al., 2021). To the best of our knowledge, we are the first to focus on predicting brain amyloid using deep learning architectures and T1-weighted structural MRIs. We know from work on related diseases (Kochunov et al., 2022) that even linear multivariate measures pick up disease effects with greater effect sizes than univariate measures, so a deep learning model could in theory produce a biomarker of atrophy that becomes abnormal or offers earlier anomaly detection and greater group differentiation than univariate measures such as hippocampal volume. As the amyloid accumulation and atrophy co-occur in the brain, it is plausible that our deep learning models could pick up on these signals to predict Aβ+. Thus, in early-stage patients who are Aβ+, the models attempt to detect any MRI-based anomalies that might separate them from healthy Aβ- subjects and combine them into a more accurate discriminator.

One potential issue with using amyloid and tau PET for molecular characterization of AD is off-target binding. While this may be a greater issue for tau PET than amyloid PET (Young et al., 2021), it is still an area of active research (Lemoine et al., 2018), because off-target binding may increase with age, affecting the SUVR metrics.

From our experiments, we can see that both deep and shallow neural networks, along with traditional classical machine learning models, showed promise in predicting amyloid positivity from standard structural brain MRI. Classical machine learning models, including XGBoost, logistic regression, and ANNs, exhibited promising balanced accuracy and F1 scores: best scores reached around 0.77. There is potential for further improvement with larger training samples and additional data modalities like Diffusion Tensor Images, which have shown significant associations with amyloid (Chattopadhyay and Singh, 2023a; Nir et al., 2023). Deep learning models, such as the 3D CNN tested, showed slightly better performance than classical machine learning models. The 2D CNN, while inferior to the 3D CNN architecture, may perform better with pre-training.

In the Alzheimer’s disease (AD) progression model proposed by Jack et al. (2013), brain amyloid typically accumulates before pervasive brain atrophy is visible on MRI. As such, predicting Aβ + may be more challenging in controls than in individuals with mild cognitive impairment (MCI) and AD, where abnormalities are already evident on both PET and MRI scans. The hybrid model achieved the highest balanced accuracy of 0.815 when incorporating hippocampal volume in the predictor set. Further enhancements may be possible by increasing the size and diversity of the training data. and incorporating data from additional cohorts. The now-standard biomarker model of Alzheimer’s disease, proposed by Jack et al. (2013), notes that structural MRI is typically one of the last biomarkers to show detectable changes - after CSF Abeta42, Amyloid PET, and CSF Tau. Because of this sequence, it is reasonable that an amyloid classifier based on T1w may not work as well in the very early stages of AD, and may work better when all of the biomarkers are somewhat elevated.

The MINiT architecture performed better than the other architecture considered—NiT. The results are promising. The performance we obtained may even improve with more training data, as the model has a large number of parameters; increasing the training dataset size may enhance model accuracy. In conclusion, the best performing models for the experiments are as summarized in Table 6.

**Table 6:** Best performing models for amyloid classification.

Model	Balanced accuracy	F1 score
Hybrid Model using Hippocampus Volume in Predictor Set	0.815	0.793
MINiT with image size (64)^3^, 6 Transformer Layers and 12 Attention Heads	0.791	0.793

The performance can improve by increasing the amount of training data.

A key goal of deep learning methods applied to neuroimaging data is that their performance remains robust even if the scanning protocol changes. In ADNI, the MRI scanning protocols do allow different scanner vendors (Siemens, Philips, and GE), but a long preparatory phase by the ADNI MRI Core was undertaken in 2004, to optimize the scan protocols for tracking of dementia, and to align the pulse sequences to the maximum possible extent across vendors. As such the training data from ADNI was from diverse scanners across the U.S., and included multiple vendors, and although the ADNI protocol was later adopted by many large scale imaging initiatives, there was still somewhat less heterogeneity in the protocols than would be seen in general. Future work will examine the use of *post-hoc* methods for MRI harmonization (Liu, 2021; Zuo et al., 2021; Komandur, 2023), to test whether this improves performance on data from new scanners and other scanning protocols.

The current biological categorization of Alzheimer’s disease commonly relies on other data sources such as amyloid- or tau-sensitive PET scans or cerebrospinal fluid (CSF) biomarkers, all of which are more invasive than structural brain MRI. While a T1w MRI-based model may benefit from the incorporation of other data sources, it offers a promising tool for benchmarking. T1w MRIs are more widely available and cost-effective than amyloid PET. Therefore, classifying amyloid positivity from T1w MRIs may help to identify participants, particularly those with MCI, for further, more intensive testing using other modalities. Prior works (Grill et al., 2019) show that the selection of biomarker criteria should be guided by the objective of enrolling individuals who are most likely to use and benefit from the intervention being studied in a specific context. As a result, our work shows the potential of ML/DL methods in MCI participants for detection of amyloid positivity before going for further more intensive testing using other modalities such as PET scans.

### 5.1 Limitations and future work

This study has limitations - notably the restricted testing on the ADNI dataset. Performance may improve with an increase in the size and diversity of the training data, by including multimodal brain MRI (Chattopadhyay and Singh, 2023a, 2023b) and by adding data from supplementary cohorts. Future work will include individuals of more diverse ancestries (John et al., 2023; Chattopadhyay and Joshy, 2024) and with various comorbidities such as vascular disease, frontotemporal dementia, and other degenerative diseases. Moreover, the sensitivity of the approach to different MRI scanning protocols and PET tracers should be examined. In the context of multisite data, harmonization methods - such as using centiloids for PET and generative adversarial networks (GANs) for MRIs - may be needed for domain adaptation. These steps may help in evaluating amyloid prediction accuracy across varied scenarios and populations. There are efforts to develop cheaper ways to measure amyloid from blood (AD Blood Tests Are Here. Now, Let’s Grapple With How to Use Them | ALZFORUM, 2024), but so far tau has been easier to measure accurately (pTau217). As these methods are developed, we hope to incorporate them into multimodal setups.

## Author’s note

Data used in preparing this article were obtained from the Alzheimer’s Disease Neuroimaging Initiative (ADNI) database (adni.loni.usc.edu/). As such, many investigators within the ADNI contributed to the design and implementation of ADNI and/or provided data but did not participate in analysis or writing of this report. A complete listing of ADNI investigators can be found at: http://adni.loni.usc.edu/wp-content/uploads/how_to_apply/ADNI_Acknowledgement_List.pdf.

## Data availability statement

Publicly available datasets were analyzed in this study. This data can be found at: https://adni.loni.usc.edu; https://www.ukbiobank.ac.uk.

## Ethics statement

Ethical approval was not required for the study involving humans in accordance with the local legislation and institutional requirements. Written informed consent to participate in this study was not required from the participants or the participants’ legal guardians/next of kin in accordance with the national legislation and the institutional requirements.

## Author contributions

TC: Conceptualization, Formal analysis, Investigation, Methodology, Project administration, Software, Writing – original draft. SO: Formal analysis, Software, Validation, Visualization, Writing – review & editing. KB: Formal analysis, Software, Validation, Visualization, Writing – review & editing. NJ: Formal analysis, Software, Validation, Visualization, Writing – review & editing. DK: Formal analysis, Software, Validation, Visualization, Writing – review & editing. JN: Formal analysis, Software, Validation, Visualization, Writing – review & editing. ST: Data curation, Writing – review & editing. GS: Writing – review & editing, Supervision. JA: Writing – review & editing, Supervision. PT: Conceptualization, Funding acquisition, Methodology, Project administration, Supervision, Writing – review & editing.

## Footnotes

^1^ http://enigma.ini.usc.edu/protocols/imaging-protocols/

^2^ https://fsl.fmrib.ox.ac.uk/fsl/fslwiki/FAST

## References

[ref1] AD Blood Tests Are Here. Now, Let’s Grapple With How to Use Them. (2024). ALZFORUM. Available at: Www.alzforum.org.

[ref2] ADNI. PET Analysis. Available at: https://adni.loni.usc.edu/methods/pet-analysis-method/pet-analysis/

[ref3] ADNI. Data types. Available at: https://adni.loni.usc.edu/data-samples/data-types/

[ref4] Alzubaidi L. Al-Amidie M. (2021). Novel transfer learning approach for medical imaging with limited labeled data. Cancer 13:1590. doi: 10.3390/cancers13071590, PMID: 33808207 PMC8036379

[ref5] Bae J. B. Lee S. Oh H. Sung J. Lee D. Han J. W.. (2023). A case-control clinical trial on a deep learning-based classification system for diagnosis of amyloid-positive Alzheimer’s disease. Psychiatry Investig. 20, 1195–1203. doi: 10.30773/pi.2023.0052, PMID: 38163659 PMC10758320

[ref6] Bi Y., “MultiCrossViT: multimodal vision transformer for schizophrenia prediction using structural MRI and functional network connectivity data,” in arXiv, (2022). Available at: http://arxiv.org/abs/2211.06726.

[ref7] Blennow K. Shaw L. M. Stomrud E. Mattsson N. Toledo J. B. Buck K.. (2019). Predicting clinical decline and conversion to Alzheimer’s disease or dementia using novel Elecsys Abeta(1-42), pTau and tTau CSF immunoassays. Sci. Rep. 9:19024. doi: 10.1038/s41598-019-54204-z, PMID: 31836810 PMC6911086

[ref8] Braak H. (2000). Vulnerability of select neuronal types to Alzheimer's disease. Ann. N. Y. Acad. Sci. 924, 53–61. doi: 10.1111/j.1749-6632.2000.tb05560.x 11193802

[ref9] Braak H. Alafuzoff I. Arzberger T. Kretzschmar H. Del Tredici K. (2006). Staging of Alzheimer disease-associated neurofibrillary pathology using paraffin sections and immunocytochemistry. Acta Neuropathol. 112, 389–404. doi: 10.1007/s00401-006-0127-z, PMID: 16906426 PMC3906709

[ref10] Braak H. Braak E. (1991). Neuropathological stageing of Alzheimer-related changes. Acta Neuropathol. 82, 239–259. doi: 10.1007/BF00308809 1759558

[ref11] Braak H. Braak E. (1997). Frequency of stages of Alzheimer-related lesions in different age categories. Neurobiol. Aging 18, 351–357. doi: 10.1016/S0197-4580(97)00056-0, PMID: 9330961

[ref12] Chattopadhyay T. Joshy N. A. (2024). Brain age analysis and dementia classification using convolutional neural networks trained on diffusion MRI: tests in Indian and north American cohorts. bioRxiv. doi: 10.1101/2024.02.04.578829v1 40039079

[ref13] Chattopadhyay T. Singh A. (2023b). Comparison of anatomical and diffusion MRI for detecting Parkinson′ s disease using deep convolutional neural network: IEEE EMBC. 1–6. 10.1109/EMBC40787.2023.10340792 38083460

[ref14] Chattopadhyay T. Singh A., (2023a). “Predicting dementia severity by merging anatomical and diffusion MRI with deep 3D convolutional neural networks.” In the 18th *international symposium on medical information processing and analysis* (Vol. 12567, pp. 90–99). SPIE.

[ref15] Cho S. H. Kim S. Choi S. M. Kim B. C. for the Alzheimer's Disease Neuroimaging Initiative (2024). ATN classification and clinical progression of the amyloid-negative Group in Alzheimer’s disease neuroimaging initiative participants. Chonnam Med. J. 60, 51–58. doi: 10.4068/cmj.2024.60.1.51, PMID: 38304128 PMC10828081

[ref16] Clark C. M. Schneider J. A. Bedell B. J. Beach T. G. Bilker W. B. Mintun M. A.. (2011). Use of florbetapir-PET for imaging beta-amyloid pathology. JAMA 305, 275–283. doi: 10.1001/jama.2010.2008, PMID: 21245183 PMC7041965

[ref17] Desikan R. S. Ségonne F. Fischl B. Quinn B. T. Dickerson B. C. Blacker D.. (2006). An automated labeling system for subdividing the human cerebral cortex on MRI scans into gyral based regions of interest. NeuroImage 31, 968–980. doi: 10.1016/j.neuroimage.2006.01.021, PMID: 16530430

[ref18] Dhinagar N. J. Thomopoulos S. I. (2023) Evaluation of transfer learning methods for detecting Alzheimer’s disease with brain MRI. In the 18th *international symposium on medical information processing and analysis* (Vol. 12567, pp. 504–513). SPIE. IEEE

[ref19] Dhinagar N. J. Thomopoulos S. I. Laltoo E. Thompson P. M. (2023). Efficiently training vision transformers on structural MRI scans for Alzheimer’s disease detection. EMBC. (pp. 1–6). IEEE. 10.1109/EMBC40787.2023.10341190 38083552

[ref20] Dufumier B. Gori P. Victor J. Grigis A. Wessa M. Brambilla P.. (2021). Contrastive learning with continuous proxy Meta-data for 3D MRI classification. MICCAI. Springer International Publishing. doi: 10.1007/978-3-030-87196-3_6

[ref21] Ezzati A. Harvey D. J. Habeck C. (2020). Predicting amyloid-β levels in amnestic mild cognitive impairment using machine learning techniques. J. Alzheimer's Dis.: JAD 73, 1211–1219. doi: 10.3233/JAD-191038, PMID: 31884486 PMC7376527

[ref22] Feng X. Provenzano F. A. Small S. A. (2022). A deep learning MRI approach outperforms other biomarkers of prodromal Alzheimer’s disease. Alzheimers Res. Ther. 14:45. doi: 10.1186/s13195-022-00985-x, PMID: 35351193 PMC8966329

[ref23] Fisch B. (2012). FreeSurfer. NeuroImage 62, 774–781. doi: 10.1016/j.neuroimage.2012.01.021, PMID: 22248573 PMC3685476

[ref24] Gelosa G. Brooks D. J. (2012). The prognostic value of amyloid imaging. Eur. J. Nucl. Med. Mol. Imaging 39, 1207–1219. doi: 10.1007/s00259-012-2108-x 22491780

[ref25] Goodfellow I. Pouget-Abadie J. Mirza M. Xu B. Warde-Farley D. Ozair S.. (2014). Generative adversarial nets. Advances in neural information processing systems, 27.

[ref26] Grill J. D. Nuño M. M. Gillen D. L. (2019). Alzheimer’s Disease Neuroimaging Initiative. Which MCI patients should be included in prodromal Alzheimer disease clinical trials? Alzheimer Dis. Assoc. Disord. 33, 104–112. doi: 10.1097/WAD.0000000000000303, PMID: 30958413 PMC6538436

[ref27] Gupta U. Lam P. K. Ver Steeg G. Thompson P. M. (2021). Improved brain age estimation with slice-based set networks. In *2021 IEEE 18th international symposium on biomedical imaging (ISBI)* (pp. 840–844).

[ref28] Hansson O. Seibyl J. Stomrud E. Zetterberg H. Trojanowski J. Q. Bittner T.. (2018). CSF biomarkers of Alzheimer’s disease concord with amyloid-β PET and predict clinical progression: a study of fully automated immunoassays in BioFINDER and ADNI cohorts. Alzheimers Dement. 14, 1470–1481. doi: 10.1016/j.jalz.2018.01.010, PMID: 29499171 PMC6119541

[ref29] Hu K. Li Y. Yu H. Hu Y. (2019). CTBP1 confers protection for hippocampal and cortical neurons in rat models of Alzheimer's disease. Neuroimmunomodulation 26, 139–152. doi: 10.1159/000500942, PMID: 31340205

[ref30] Huang G. Liu Z. van der Maaten L. Weinberger K. Q. (2017). Densely connected convolutional networks. In Proceedings of the IEEE conference on computer vision and pattern recognition. 4700–4708.

[ref31] Jack C. R. Jr. Bennett D. A. Blennow K. Carrillo M. C. Dunn B. Haeberlein S. B.. (2018). NIA-AA research framework: toward a biological definition of Alzheimer's disease. Alzheimers Dement. 14, 535–562. doi: 10.1016/j.jalz.2018.02.018, PMID: 29653606 PMC5958625

[ref32] Jack C. R. Jr. Knopman D. S. Jagust W. J. Petersen R. C. Weiner M. W. Aisen P. S.. (2013). Tracking pathophysiological processes in Alzheimer's disease: an updated hypothetical model of dynamic biomarkers. Lancet. Neurol. 12, 207–216. doi: 10.1016/S1474-4422(12)70291-0, PMID: 23332364 PMC3622225

[ref33] Jang J. Hwang D. (2022). M3T: Three-dimensional medical image classifier using multi-plane and multi-slice transformer, 20718–20729.

[ref34] Jin Y. DuBois J. Zhao C. Zhan L., (2023). “Brain MRI to PET synthesis and amyloid estimation in Alzheimer's disease via 3D multimodal contrastive GAN.” In *International workshop on machine learning in medical imaging* (pp. 94–103). Cham: Springer Nature.

[ref35] John J. P. Joshi H. Sinha P. Harbishettar V. Tripathi R. Cherian A. V.. (2023). India ENIGMA initiative for Global Aging & Mental Health–a globally coordinated study of brain aging and Alzheimer’s disease. Alzheimers Dement. 19:e076394. doi: 10.1002/alz.076394

[ref36] Johnson G. V. Hartigan J. A. (1999). Tau protein in normal and Alzheimer's disease brain: an update. J. Alzheimers Dis. 1, 329–351. doi: 10.3233/JAD-1999-14-512, PMID: 12214129

[ref37] Kim H. E. Cosa-Linan A. Santhanam N. Jannesari M. Maros M. E. Ganslandt T. (2022). Transfer learning for medical image classification: a literature review. BMC Med. Imaging 22:69. doi: 10.1186/s12880-022-00793-7, PMID: 35418051 PMC9007400

[ref38] Kim J. P. Kim J. Jang H. Kim J. Kang S. H. Kim J. S.. (2021). Predicting amyloid positivity in patients with mild cognitive impairment using a radiomics approach. Sci. Rep. 11:6954. doi: 10.1038/s41598-021-86114-4, PMID: 33772041 PMC7997887

[ref39] Kim S. Lee P. Oh K. T. Byun M. S. Yi D. Lee J. H.. (2021). Deep learning-based amyloid PET positivity classification model in the Alzheimer’s disease continuum by using 2-[^18^F] FDG PET. Eur J Nucl Med Mol Imag. Res. 11:56. doi: 10.1186/s13550-021-00798-3 PMC8192639 34114091

[ref40] Kingma D. Ba J. (2015). Adam: A method for stochastic optimization: ICLR.

[ref41] Klunk W. E. Engler H. Nordberg A. Wang Y. Blomqvist G. Holt D. P.. (2004). Imaging brain amyloid in Alzheimer's disease with Pittsburgh compound-B. Ann. Neurol. 55, 306–319. doi: 10.1002/ana.20009 14991808

[ref42] Kochunov P. Fan F. Ryan M. C. Hatch K. S. Tan S. Jahanshad N.. (2022). Translating ENIGMA schizophrenia findings using the regional vulnerability index: association with cognition, symptoms, and disease trajectory. Hum. Brain Mapp. 43, 566–575. doi: 10.1002/hbm.25045, PMID: 32463560 PMC8675428

[ref43] Koivunen J. Karrasch M. Scheinin N. M. Aalto S. Vahlberg T. Någren K.. (2012). Cognitive decline and amyloid accumulation in patients with mild cognitive impairment. Dement. Geriatr. Cogn. Disord. 34, 31–37. doi: 10.1159/000341580 22868352

[ref44] Komandur D., (2023). Unsupervised harmonization of brain MRI using 3D CycleGANs and its effect on brain age prediction. *19th International symposium on medical information processing and analysis* (SIPAIM) (pp. 1–5). IEEE.

[ref45] Lam P. Zhu A. H. (2020). 3-D grid-attention networks for interpretable age and Alzheimer’s disease prediction from structural MRI. arXiv preprint arXiv:2011.09115.

[ref46] Landau S. M. Breault C. Joshi A. D. Pontecorvo M. Mathis C. A. Jagust W. J.. (2013). Amyloid-β imaging with Pittsburgh compound B and florbetapir: comparing radiotracers and quantification methods. J. Nucl. Med. 54, 70–77. doi: 10.2967/jnumed.112.109009, PMID: 23166389 PMC3747730

[ref47] Landau S. M. Thomas B. A. (2014). Amyloid PET imaging in Alzheimer’s disease: a comparison of three radiotracers. Eur. J. Nucl. Med. Mol. Imaging 41, 1398–1407. doi: 10.1007/s00259-014-2753-3, PMID: 24647577 PMC4055504

[ref48] Lemoine L. Leuzy A. Chiotis K. Rodriguez-Vieitez E. Nordberg A. (2018). Tau positron emission tomography imaging in tauopathies: the added hurdle of off-target binding. Alzheimers Dement (Amst). 10, 232–236. doi: 10.1016/j.dadm.2018.01.007 29780868 PMC5956931

[ref49] Li J. (2022). Transforming medical imaging with transformers? A comparative review of key properties, current progresses, and future perspectives. arXiv 2206:01136. 10.1016/j.media.2023.102762 PMC10010286 36738650

[ref50] Liu M., (2021). Style transfer using generative adversarial networks for multi-site mri harmonization. In *Medical Image Computing and Computer Assisted Intervention–MICCAI 2021: 24th International Conference*, Strasbourg, France, September 27–October 1, 2021, Proceedings, Part III 24 (pp. 313–322). Springer International Publishing. 10.1007/978-3-030-87199-4_30 PMC9137427 35647615

[ref51] Lu B. Li H.-X. Chang Z.-K. Li L. Chen N. X. Zhu Z. C.. (2022). A practical Alzheimer disease classifier via brain imaging-based deep learning on 85,721 samples. J. Big Data 9:101. doi: 10.1186/s40537-022-00650-y

[ref52] Masters C. L. Selkoe D. J. (2012). Biochemistry of amyloid β-protein and amyloid deposits in Alzheimer disease. Cold Spring Harb. Perspect. Med. 2:a006262. doi: 10.1101/cshperspect.a006262, PMID: 22675658 PMC3367542

[ref53] Matsoukas C., “Is it time to replace CNNs with transformers for medical images?” (2021). Available at: http://arxiv.org/abs/2108.09038

[ref54] Morid M. A. Borjali A. Fiol G. D. (2021). A scoping review of transfer learning research on medical image analysis using ImageNet. Comput. Biol. Med. 128:104115. doi: 10.1016/j.compbiomed.2020.104115, PMID: 33227578

[ref55] Nelson P. T. Jicha G. A. Schmitt F. A. Liu H. Davis D. G. Mendiondo M. S.. (2007). Clinicopathologic correlations in a large Alzheimer disease center autopsy cohort: neuritic plaques and neurofibrillary tangles" do count" when staging disease severity. J. Neuropathol. Exp. Neurol. 66, 1136–1146. doi: 10.1097/nen.0b013e31815c5efb 18090922 PMC3034246

[ref56] Nir T. M. Villalón-Reina J. E. Salminen L. E. (2023). Cortical microstructural associations with CSF amyloid and pTau. Mol. Psychiatry 1–12. 38092890 10.1038/s41380-023-02321-7 PMC11116103

[ref57] Okello A. Koivunen J. Edison P. Archer H. A. Turkheimer F. E. Någren K.. (2009). Conversion of amyloid positive and negative MCI to AD over 3 years: an 11C-PIB PET study. Neurology 73, 754–760. doi: 10.1212/WNL.0b013e3181b23564, PMID: 19587325 PMC2830881

[ref58] Pan Y. Liu M. Lian C. Zhou T. Xia Y., “Synthesizing missing PET from MRI with cycle-consistent generative adversarial networks for Alzheimer’s disease diagnosis,” *21st International Conference, Granada, Spain, Proceedings*, Part 11072. (2018). 10.1007/978-3-030-00931-1_52 PMC8336606 34355223

[ref59] Petersen R. C. Smith G. E. Waring S. C. Ivnik R. J. Tangalos E. G. Kokmen E. (1999). Mild cognitive impairment: clinical characterization and outcome. Arch. Neurol. 56, 303–308. doi: 10.1001/archneur.56.3.303 10190820

[ref60] Petrone P. M. Casamitjana A. (2019). Prediction of amyloid pathology in cognitively unimpaired individuals using voxel-wise analysis of longitudinal structural brain MRI. Alzheimers Res. Ther. 11:72. doi: 10.1186/s13195-019-0526-8, PMID: 31421683 PMC6698344

[ref61] Qu C. Zou Y. Dai Q. Ma Y. He J. Liu Q.. (2021). Advancing diagnostic performance and clinical applicability of deep learning-driven generative adversarial networks for Alzheimer's disease. Psychoradiology 1, 225–248. doi: 10.1093/psyrad/kkab017, PMID: 38666217 PMC10917234

[ref62] Revised Again: Alzheimer’s Diagnostic Criteria Get Another Makeover. (2023) ALZFORUM. Available at: Www.alzforum.org.

[ref63] Rowe C. C. Ellis K. A. Rimajova M. Bourgeat P. Pike K. E. Jones G.. (2010). Amyloid imaging results from the Australian imaging, biomarkers and lifestyle (AIBL) study of aging. Neurobiol. Aging 31, 1275–1283. doi: 10.1016/j.neurobiolaging.2010.04.007 20472326

[ref64] Shan G. Bernick C. Caldwell J. Z. K. Ritter A. (2021). Machine learning methods to predict amyloid positivity using domain scores from cognitive tests. Sci. Rep. 11:4822. doi: 10.1038/s41598-021-83911-9, PMID: 33649452 PMC7921140

[ref65] Singla A. Zhao Q. do D. K. Zhou Y. Pohl K. M. Adeli E. (2022). Multiple Instance Neuroimage Transformer. Predictive Intelligence in Medicine: 5th International Workshop, MICCAI PRIME 13564, 36–48. doi: 10.1007/978-3-031-16919-9_4 PMC9629332 36331280

[ref66] Son H. J. Oh J. S. Oh M. Kim S. J. Lee J. H. Roh J. H.. (2020). The clinical feasibility of deep learning-based classification of amyloid PET images in visually equivocal cases. Eur. J. Nucl. Med. Mol. Imaging 47, 332–341. doi: 10.1007/s00259-019-04595-y, PMID: 31811343

[ref67] SPM12 software - Statistical Parametric Mapping. (2014). Functional Imaging Laboratory. Available at: https://www.fil.ion.ucl.ac.uk/spm/software/spm12/

[ref68] Sudlow C. Gallacher J. Allen N. Beral V. Burton P. Danesh J.. (2015). UK biobank: an open access resource for identifying the causes of a wide range of complex diseases of middle and old age. PLoS Med. 12:e1001779. doi: 10.1371/journal.pmed.1001779, PMID: 25826379 PMC4380465

[ref69] Tarasoff-Conway J. M. Carare R. O. Osorio R. S. Glodzik L. Butler T. Fieremans E.. (2015). Clearance systems in the brain implications for Alzheimer disease. Nat. Rev. Neurol. 11, 457–470. doi: 10.1038/nrneurol.2015.119, PMID: 26195256 PMC4694579

[ref70] Thompson P. M. (2007). Tracking Alzheimer's disease. Ann. N. Y. Acad. Sci. 1097, 183–214. doi: 10.1196/annals.1379.017, PMID: 17413023 PMC3197831

[ref71] Thompson P. M. Hayashi K. M. Sowell E. R. Gogtay N. Giedd J. N. Rapoport J. L.. (2004). Mapping cortical change in Alzheimer's disease, brain development, and schizophrenia. NeuroImage 23, S2–S18. doi: 10.1016/j.neuroimage.2004.07.071, PMID: 15501091

[ref72] van Dyck C. H. Swanson C. J. Aisen P. Bateman R. J. Chen C. Gee M.. (2023). Lecanemab in early Alzheimer's disease. N. Engl. J. Med. 388, 9–21. doi: 10.1056/NEJMoa2212948 36449413

[ref73] Van Erp T. G. Hibar D. P. (2016). Subcortical brain volume abnormalities in 2028 individuals with schizophrenia and 2540 healthy controls via the ENIGMA consortium. Mol. Psychiatry 21, 547–553. doi: 10.1038/mp.2015.63, PMID: 26033243 PMC4668237

[ref74] van Erp T. G. M. Walton E. Hibar D. P. Schmaal L. Jiang W. Glahn D. C.. (2018). Cortical brain abnormalities in 4474 individuals with schizophrenia and 5098 control subjects via the enhancing neuro imaging genetics through meta analysis (ENIGMA) consortium. Biol. Psychiatry 84, 644–654. doi: 10.1016/j.biopsych.2018.04.023, PMID: 29960671 PMC6177304

[ref75] Veitch D. Weiner M. W. Aisen P. S. Beckett L. A. Cairns N. J. Green R. C.. (2019). Understanding disease progression and improving Alzheimer's disease clinical trials: recent highlights from the Alzheimer's disease neuroimaging initiative. Alzheimers Dement. 15, 106–152. doi: 10.1016/j.jalz.2018.08.005, PMID: 30321505

[ref76] Villemagne V. L. Burnham S. Bourgeat P. Brown B. Ellis K. A. Salvado O.. (2013). Amyloid β deposition, neurodegeneration, and cognitive decline in sporadic Alzheimer’s disease: a prospective cohort study. Lancet Neurol. 12, 357–367. doi: 10.1016/S1474-4422(13)70044-9 23477989

[ref77] Villemagne V. L. Pike K. E. Chételat G. Ellis K. A. Mulligan R. S. Bourgeat P.. (2011). Longitudinal assessment of Aβ and cognition in aging and Alzheimer disease. Ann. Neurol. 69, 181–192. doi: 10.1002/ana.22248, PMID: 21280088 PMC3045039

[ref78] Wang T. Lei Y. Fu Y. Wynne J. F. Curran W. J. Liu T.. (2020). A review on medical imaging synthesis using deep learning and its clinical applications. J. Appl. Clin. Med. Phys. 22, 11–36. doi: 10.1002/acm2.13121, PMID: 33305538 PMC7856512

[ref79] Willemink M. J. Roth H. R. Sandfort V. (2022). Toward foundational deep learning models for medical imaging in the new era of transformer networks. Radiol. Artif. Intell. 4:e210284. doi: 10.1148/ryai.210284 36523642 PMC9745439

[ref80] World Health Organization. “Dementia,” (2022). Available at: https://www.who.int/news-room/fact-sheets/detail/dementia.

[ref81] Yasuno F. Kazui H. Morita N. Kajimoto K. Ihara M. Taguchi A.. (2017). Use of T1-weighted/T2-weighted magnetic resonance ratio to elucidate changes due to amyloid β accumulation in cognitively normal subjects. NeuroImage: Clinical 13, 209–214. doi: 10.1016/j.nicl.2016.11.029, PMID: 28003959 PMC5157788

[ref82] Youden W. J. (1950). Index for rating diagnostic tests. Cancer 3, 32–35. doi: 10.1002/1097-0142(1950)3:1<32::AID-CNCR2820030106>3.0.CO;2-3 15405679

[ref83] Young C. B. Landau S. M. Harrison T. M. Poston K. L. Mormino E. C. (2021). Influence of common reference regions on regional tau patterns in cross-sectional and longitudinal [18F]-AV-1451 PET data. NeuroImage 243:118553. doi: 10.1016/j.neuroimage.2021.118553 34487825 PMC8785682

[ref84] Zhou T. Ye X. Y. Lu H. L. Zheng X. Qiu S. Liu Y. C. (2022). Dense convolutional network and its application in medical image analysis. Biomed. Res. Int. 2022, 1–22. doi: 10.1155/2022/2384830 PMC9060995 35509707

[ref85] Zhuang F. Qi Z. Duan K. Xi D. Zhu Y. Zhu H.. (2020). A comprehensive survey on transfer learning. Proc. IEEE 109, 43–76. doi: 10.1109/JPROC.2020.3004555

[ref86] Zuo L. Dewey B. E. Carass A. Liu Y. He Y. Calabresi P. A., (2021). Information-based disentangled representation learning for unsupervised MR harmonization. In the *international conference on information processing in medical imaging* (pp. 346–359). Cham: Springer International Publishing.

