# Supplementary material for: Comparison of deep learning architectures for predicting amyloid positivity in Alzheimer’s disease, mild cognitive impairment, and healthy aging, from T1-weighted brain structural MRI
Source: Front Neurosci. 2024 Jul 2;18:1387196. doi: 10.3389/fnins.2024.1387196 (PMC11250587; doi:10.3389/fnins.2024.1387196)
Supplement: Supplementary file 1 [file Data_Sheet_1.PDF]

Supplementary Material

Comparison of Deep Learning Architectures for Predicting Amyloid Positivity in Alzheimer's Disease, Mild Cognitive Impairment, and Healthy Aging, from T1-weighted Brain Structural MRI

Tamoghna Chattopadhyay[1*], Saket S. Ozarkar[1], Ketaki Buwa[1], Neha Ann Joshy[1], Dheeraj Komandur[1], Jayati Naik[1], Sophia I. Thomopoulos[1], Greg Ver Steeg[2], Jose Luis Ambite[3], Paul M. Thompson[1*], for the Alzheimer's Disease Neuroimaging Initiative (ADNI)

[1]Imaging Genetics Center, Mark and Mary Stevens Neuroimaging and Informatics Institute, Keck School of Medicine, University of Southern California, Marina del Rey, CA, United States

[2]University of California, Riverside, CA, United States

[3]Information Sciences Institute, University of Southern California, Marina del Rey, CA, United States

Correspondence: Tamoghna Chattopadhyay, tchattop@usc.edu,
Paul M. Thompson, pthomp@usc.edu

Table of Contents

1. SubjID Explanation ……………………………………………………………2
2. SubjIDs of subjects in Training Dataset ………………………………………………2
3. SubjIDs of subjects in Validation Dataset ……………………………………………..4
4. SubjIDs of subjects in Testing Dataset ………………………………………………...5
5. Transfer Learning Experiments……………………………………………………...6
6. Transfer Learning Results and Discussions…………………………………………….6
7. 3D DenseNet121 Experiment and Results………………………………………………...8

1. T1-weighted Image ID Explanation

All the Subjects from ADNI which have been used to make the training, validation and test sets are mentioned here. These are the T1-weighted Image IDs in the ADNI database, which can be used to replicate the results from the experiments.

2. T1-weighted Image ID of subjects in Training Dataset

241350,242895,373133,649030,291874,326600,291876,334105,291880,353879,470395,225356,
362927,500242,229307,362929,775697,221959,321318,510016,222697,222705,451354,782893,
250649,255409,392163,258686,260229,398197,765035,260237,398199,260241,280582,261069,
399888,765011,765034,280562,418775,285129,729600,418843,729590,297035,424729,312646,
782900,349888,374467,379928,384094,291886,299336,498002,291898,503715,395048,254756,
388308,250665,255415,263742,272720,431684,278119,431686,844759,278123,277673,412944,
288883,291888,300940,322305,350442,327046,392288,392426,392428,392430,291908,291914,
848326,382260,506289,254768,525976,258625,398209,544623,306746,337588,351980,764998,
293753,250657,764991,250637,391062,260245,398263,267757,412262,765014,270050,431682,
280570,418845,727161,283956,506302,295949,312648,306760,430885,765007,322531,340498,
466136,369218,510015,326616,222427,222569,327080,388315,398122,775721,272695,430887,
281397,282674,649033,415579,394747,421248,295877,430247,848405,296298,848419,848403,
331714,395090,775701,338650,727835,466139,844783,358349,474235,236978,502081,272728,
722154,267745,722151,413089,285480,431819,422842,729639,295885,312757,782878,315320,
335416,339153,349550,353184,358357,414458,432901,502102,414462,200364,729587,570703,
300270,353875,362967,474220,377751,431749,248611,398145,258684,392127,544749,260231,
557511,398114,557534,267763,729648,315322,321232,782899,331853,229345,498015,241356,
727679,296415,384806,544729,250623,388382,258662,398276,291049,420257,300513,423876,
800065,321316,498004,506275,377881,384096,488742,265254,399883,280662,431690,729740,
498024,297655,443314,300287,442447,443076,331857,392143,352005,362973,369614,377079,
222749,729721,241346,238666,242899,296282,261065,267747,270063,764996,290963,430249,
293681,222761,222767,321314,241397,377889,248638,388404,557504,431753,293689,421239,
306753,305486,326634,326636,451352,848404,338586,845375,350342,477210,369717,498008,
350731,369719,388021,222775,358477,466165,222779,351990,468860,358487,470401,270069,
416005,765437,267767,272814,408336,274508,412940,300508,765020,321264,440493,326529,
388409,297290,297294,557527,272652,281432,414365,288881,300946,321304,766468,765039,
349820,349866,379548,510017,337444,369385,498060,503720,331703,222783,350477,222787,
349302,222791,222795,222809,349294,260227,412390,261059,267759,272816,273516,431802,
312842,300293,306699,312651,321451,444081,379938,222841,370015,844389,222851,363026,
474218,258657,844390,417074,788071,267771,722150,270056,415836,883332,408347,722153,
285472,420261,788058,321453,439902,349822,332577,332567,222458,350481,233460,377161,
506287,241370,557532,722827,466159,722110,283936,418704,281430,415838,764970,290953,
423847,326638,374612,388415,222916,241394,510050,254754,391090,254748,414377,544743,
258623,263676,420287,300950,765013,326594,353186,391092,222931,358194,474192,222933,
353869,222935,225376,411327,432425,439320,321466,321468,451370,331861,451350,466151,
337570,451364,338582,339188,340506,392329,488705,414563,498048,414567,498054,414525,

498064,414527,498081,414531,502109,544727,392161,844798,217737,294865,394814,411334,
254758,388417,852306,261084,399906,267783,267743,274510,423853,424751,845421,434253,
305405,432881,845431,379940,544721,392331,557539,379942,557537,728497,218327,217750,
370060,222954,322256,222973,425937,228527,370064,232862,232860,235655,373105,235659,
363266,250647,389147,258680,258666,394745,258660,398315,425935,283922,430891,291927,
305613,222998,350726,570692,223008,223011,728508,263730,273518,722108,274506,729650,
314561,322260,502129,398319,765032,217765,218358,218371,218752,218790,254802,391094,
721347,392131,765029,398321,721278,283946,417075,285123,349898,377083,500230,379955,
388433,544760,436810,300282,223013,223015,351550,223019,389149,570695,296310,248632,
272818,421255,281845,837076,315324,313649,451331,377163,238655,388439,241348,882729,
272716,420283,415840,283932,417077,727763,290955,297891,420265,432422,434255,305484,
312653,434932,371245,322303,440930,326520,358062,384098,388447,388449,765435,506277,
411351,411353,498079,234654,498066,765429,255435,398327,544716,258627,398329,263752,
286643,280574,305428,421257,765545,418779,721352,314559,488790,322292,414488,502107,
414490,414492,765546,510018,374623,229335,498050,238646,377018,498068,238651,373219,
241798,506279,241342,379959,498077,525918,248677,388458,544750,260243,398331,261083,
398333,765537,273513,431805,729589,296425,417090,300952,308100,322301,451336,442453,
466131,342430,415949,477222,371403,494160,371416,488748,377026,382256,510019,506293,
296545,296511,349583,349828,398096,765547,296541,296549,488802,296423,322467,446704,
326640,782876,470407,349800,357958,403739,765536,229333,248609,376856,765617,248669,
291000,432193,291936,765031,322469,443080,357854,376832,544713,377879,388027,765611,
391059,416077,598368,248625,384804,322359,771168,375638,402028,845550,468856,468854,
388031,223044,351400,223055,349700,371573,477228,238653,255433,255421,398343,557513,
399890,312636,312678,431631,765624,322519,436840,337565,374643,377885,382254,844897,
223065,352003,494158,223073,223075,223077,225398,241364,392145,241366,248663,254775,
255437,261077,267769,510014,270045,274671,286646,363362,845647,313662,351376,223219,
223240,222683,335942,451342,223251,223259,340482,223263,338713,223267,350252,223271,
598493,223279,351436,371622,241377,248636,248634,389153,258717,598183,598175,598211,
599529,275763,599517,598181,283938,598184,285484,788073,286083,296336,296327,598189,
312749,322625,440505,326503,340527,377166,477203,258699,286652,413099,817482,223283,
371678,223287,223291,371694,223297,371706,544624,223326,370075,765615,223345,371708,
765623,248679,254822,557518,403744,263689,431650,263680,403756,263682,403761,721350,
267765,412935,721297,267773,404321,270031,403751,722091,270054,776140,285131,285488,
800068,288879,915502,291929,436823,312686,800059,312680,315370,322646,339187,468866,
349543,421245,377168,502104,502098,223357,223362,223367,389157,241401,392129,263728,
431677,274514,349694,414450,374699,387866,391070,357856,223371,371815,223373,371817,
232854,374701,254760,398347,557535,263732,398103,417106,288885,293721,423861,846730,
430241,305480,431635,846433,915496,297011,297014,846816,340553,223379,451356,223385,
466133,844923,225425,374705,775722,229328,374707,541293,241354,258658,398126,265252,
846813,300278,342548,296532,350369,223458,883328,296345,388618,296347,392137,296349,
260235,261079,283954,283926,295863,788072,297041,275408,290965,423834,300298,424745,
305408,766469,821698,362911,362900,498062,371986,371988,223481,282728,225354,431761,
846736,415846,291002,431629,291870,425931,727661,281847,280917,721345,352008,506273,
297275,297281,236974,411362,498058,242878,258635,544738,254787,766198,255413,391119,
254785,398345,574597,415901,267741,281425,413083,281407,415848,729620,296584,424739,
312655,544733,326508,274707,225427,340537,229341,250621,398352,254811,398354,285119,

312845,312849,451334,335975,260275,398370,557512,270025,402020,666341,281837,413091,
282672,415850,283924,285133,300300,305417,322357,326506,776143,376906,502131,382272,
297319,728504,544754,467242,915538,223495,250659,585820,398372,544715,255411,255431,
398374,398376,402024,847271,285127,418787,291900,421272,312640,444089,432199,312718,
448292,322917,448288,322919,448296,451362,782885,337437,349916,353885,357766,358086,
414569,414571,500240,432918,414573,280569,280591,299241,414373,299365,280595,297912,
223497,314522,431643,766211,223507,223509,466141,349521,466143,223515,349288,477226,
223523,223525,474216,225593,286635,290959,423839,312674,291878,295859,424733,296406,
295861,424960,300250,430896,305440,312658,434263,766291,312672,312864,322932,451368,
322939,358509,477224,451366,225431,411325,312705,241399,263726,398120,848306,267779,
402036,402038,403759,272812,408343,666338,848307,234666,498070,297599,385960,398386,
261071,398147,402040,267761,399896,270018,402042,280550,280552,413081,280572,283934,
417099,400998,498027,291894,431775,300302,308045,474228,765419,326532,498017,474208,
350510,349560,414602,351548,362848,432883,498013,414452,414456,766262,477232,398143,
557531,263754,399898,403733,280558,722826,283942,413097,290940,421282,291884,423874,
729598,297888,424053,305422,430239,431779,766278,305621,432180,766303,337552,349914,
377193,384808,544732,388925,217978,258682,399861,265203,281843,295879,377195,337453,
451348,788059,994520,338594,442459,218005,337467,782889,357731,297876,353188,468862,
204817,357752,442457,544730,258692,398394,261075,915544,265269,267777,431706,766394,
766393,418795,285903,729588,296355,421286,765027,288887,431646,300252,766474,766389,
420267,296410,424968,305433,437694,305446,438131,300256,432201,305449,326526,451340,
326584,451358,322345,388927,544751,219111,416102,297903,297904,222640,788064,431490,
260223,305539,334124,342384,223530,557523,223535,403766,517389,248630,248654,388931,
250661,254777,398398,254764,392165,254770,265201,270067,766388,295881,384092,557516,
384777,225433,242893,223539,236982,398193,398409,696383,412276,729741,261061,414585,
272646,412392,270042,766473,275760,368981,729735,729725

3. T1-weighted Image ID of subjects in Validation Dataset:

234658,412309,261073,399900,425939,286639,729728,312847,436842,765045,764969,235638,
291917,242887,369214,291923,270043,283929,434261,285469,296530,377766,349246,275414,
222736,272772,272700,283940,418847,222684,286683,241381,388351,506368,394756,296413,
417097,337435,544734,729601,351339,374561,544717,297648,300289,788713,222751,401002,
416003,295883,300955,288895,288899,421241,294869,296404,312727,338584,666345,340529,
222781,721318,764968,305601,312861,384090,399863,418550,765040,422844,377764,258719,
274516,370009,377010,379936,236970,294815,411360,255419,398283,254783,254821,294830,
349537,388240,544720,275417,430243,294859,470399,431755,321464,337441,358248,488707,
394754,498046,414565,388029,848497,286644,222947,229343,281434,771128,411322,267781,
418777,388431,721319,254809,727164,349886,363278,844881,349516,544758,430893,274667,
432954,371899,228531,557510,274518,335394,295940,229337,377016,727733,285452,326580,
334061,466163,363334,371441,337439,765535,844929,431759,350690,470397,394766,765618,
765612,296805,326534,327041,398341,570688,477234,223061,392139,265199,223067,223069,
374660,305605,322550,334169,598498,241403,255405,594041,296329,800066,420254,431626,
423836,434257,315318,388531,402030,388019,488745,498052,417108,393650,280554,845623,
424958,846467,466153,238649,260233,288877,451372,223462,229339,286093,291051,414371,
500226,389161,846734,494162,300258,388911,470403,228507,389167,296402,766200,442455,

389169,248656,417104,440499,306758,771152,544741,351341,468864,817481,847257,260225,263740,296528,331859,392333,843949,414605,506290,544748,280566,280593,223503,223513,223519,357762,421278,766205,451338,223527,339147,488820,377185,258655,270037,414448,848304,418791,848308,966279,477206,376955,263683,267753,403754,272692,300297,305615,322957,494168,388923,403741,281889,727794,729599,225435,238647,258664,277041,280560,312866,544728,544761,544725,416106,424049,248626,258653,765420,431719,277667,294867,434293,375627

4. T1-weighted Image ID of subjects in Test Dataset:

338715,764973,258694,525980,299363,408327,291892,299332,244937,326606,392270,392272,217663,398207,398261,775723,263678,848324,413095,844786,424966,431855,451346,474232,411336,727660,439312,439314,474236,432910,414460,340931,506278,263685,335396,451360,327035,374563,727686,364667,727683,222747,395167,225559,299280,338588,205483,242900,288898,222773,270023,295865,222777,401951,424747,434934,771151,398149,297292,416007,300948,300275,222811,335382,370011,374610,222825,349248,377159,782882,412286,283918,337554,294805,248665,404304,267751,293685,431815,466157,294832,461144,557536,358196,470409,494166,312676,326588,326484,442451,337589,339185,502126,392325,432908,274703,267755,300942,314520,358022,371729,281841,466145,416035,335406,254779,296308,388435,291903,291896,448284,765016,722155,430883,322288,342433,358048,377052,297310,492184,229326,411357,765543,727735,305482,765544,498006,248650,392135,557540,417095,717789,342390,466161,363336,477230,371434,500222,388460,474214,506317,296583,223039,470405,308047,434285,439904,223058,371571,296321,382258,506318,371250,223063,225403,322529,306756,598363,349298,598404,300515,377165,379965,248648,277671,280556,598186,598212,223347,342493,393658,398117,261063,274669,421265,782890,308102,437408,466169,461137,363221,389155,349300,474870,255417,258690,267749,223369,474222,474190,263736,223376,340567,422851,351380,254781,350327,517388,369381,788060,223483,276573,285905,766209,502103,431800,389165,322343,915537,424055,305603,440497,440934,223493,357757,388815,254772,412307,432789,414575,488738,223521,362861,362865,492186,766236,312736,312843,771138,382247,394752,270065,379550,270027,415852,294875,296408,322951,340500,349785,350508,414454,372037,766273,766268,412942,727165,431777,544726,280564,436808,451332,297874,467240,300962,431478,265267,402050,270058,403749,431811,291882,766395,430245,312660,782881,369082,297899,427957,277669,398112,296357,398400,323002,729722,241396,312638

5. Transfer Learning Experiments:

For pre-training, we used a traditional supervised learning approach with labeled training data. The initial network state was defined by using the above 3D-CNN architecture to predict the sex of the subjects from T1w scans from the UK Biobank cohort. The sex classification task has been used with success as a pre-training task for AD classification [29,33], even outperforming age estimation as a pre-training task.

The 3D-CNN was trained for 40 epochs with the Adam (with weight decay) optimizer with a learning rate of $1x10^{-4}$ and a learning rate scheduler. This trained model was fine-tuned to predict Aβ+ using three methods: (1) the model was used with the trained weights; (2) the model's last two layers were unfrozen; and (3) the model was fine-tuned end to end. For all three methods, the batch size was set to 6 and the model was trained until the validation loss did not improve for 10 consecutive epochs. We also wanted to understand whether the amount of data in the pretraining task affected downstream task performance. Accordingly, the UK Biobank dataset was divided into 8 batches of 2,000 images each, while training the model. Weights for each of these models were stored as the starting weights for downstream tasks. Thus, while fine tuning, the accuracies were calculated for all 8 sets of initial starting weights.

The same method was used for pre-training on age prediction, i.e., the initial state of the network was defined by using the above 3D CNN architecture to predict the age of healthy controls from the UK Biobank cohort, based on their T1w scans. The loss function in this case was mean squared error. The fine-tuning for downstream tasks was the same as in the prior experiments, with the same hyperparameter values. The model was evaluated using balanced accuracy and F1 Score, using a threshold obtained with Youden's Index. Each model was run three times, and the average value reported.

6. Transfer Learning Results and Discussions:

For the transfer learning experiments, the pre-training algorithms did not boost the classification accuracy (**Figure 1).** When predicting Aβ positivity, models pre-trained on sex classification in the UK Biobank dataset achieved a balanced accuracy of approximately 0.690. The accuracy decreased to around 0.660 when the model was pre-trained on age prediction in the UK Biobank dataset. For the pretraining task of UK Biobank sex classification, the highest accuracy observed was 0.531 when all the data was used, and all layers of the model were frozen, i.e., a random chance performance. However, when the bottom two layers were unfrozen, the highest average balanced accuracy reached 0.660 with 12,000 images used in pretraining. In the case of end-to-end training, the average balanced accuracy reached 0.690 with 10,000 data points from the UK Biobank in the preliminary training task.

When using the UK Biobank brain age prediction network without any adaptation (all layers frozen), the performance was at chance level, yielding an average balanced accuracy of around 0.457. Unfreezing the bottom two layers improved accuracy, with the highest achieved at 0.650 using 12,000 images in pretraining. End-to-end training further increased accuracy to 0.678 with 16,000 images from the UK Biobank in the preliminary training task. Linear regression models

were employed using the values of balanced accuracy and the number of data points, the slopes and p-values are as presented in **Table 1**.

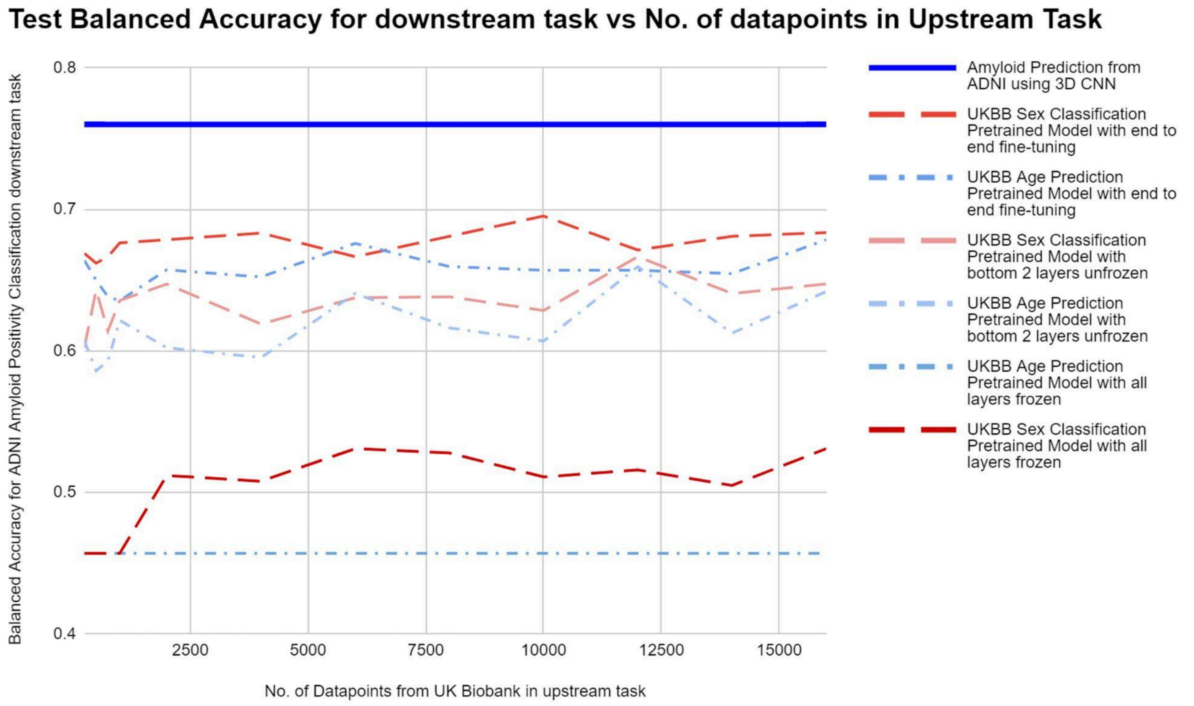

Figure 1. Plot of ADNI test set Balanced Accuracy vs % of training scans in pretraining from UK Biobank data. The topmost line represents the balanced accuracy when the 3D CNN model is trained from scratch on ADNI data, without any pre-training.

Table 1. Slopes and p-values for linear regression model for balanced accuracy vs. number of data points.

**Experiment**	**Slope**	**p-value**
UKBB Sex Classification Pretrained Model with end to end fine-tuning	8.948e-007 ± 4.510e-007	0.0754
UKBB Age Prediction Pretrained Model with end to end fine-tuning	1.166e-006 ± 6.010e-007	0.081
UKBB Sex Classification Pretrained Model with bottom 2 layers unfrozen	1.601e-006 ± 7.998e-007	0.0732
UKBB Age Prediction Pretrained Model with bottom 2 layers unfrozen	2.433e-006 ± 9.661e-007	0.0305
UKBB Age Prediction Pretrained Model with all layers frozen	-	-
UKBB Sex Classification Pretrained Model with all layers frozen	3.976e-006 ± 1.189e-006	0.0074

Four broad conclusions may be drawn from the pre-training results (**Figure 1**). First, pre-training on a large dataset did not enhance downstream task accuracy in this scenario. This can be seen by the p-values which are non significant in the cases with best balanced accuracy. Second, the amount of data used in pre-training did not significantly impact the accuracy of the downstream amyloid positivity prediction task. Third, models yielded similar results with minor variations in balanced accuracy after using 2,000 data points in the pretraining task. Lastly, pretraining to predict sex from T1w MRIs of the UK Biobank yielded marginally better accuracy in the downstream task of amyloid prediction, compared to pretraining for age prediction from T1w MRIs.

The transfer learning experiments suggest that accuracy is higher without pre-training, at least in the set-up that we examined. This may be because the UK Biobank consists primarily of healthy subjects, and may not offer optimal predictive features for amyloid detection in the Alzheimer's Disease Neuroimaging Initiative (ADNI) dataset. Based on our experiments, pre-training was not always beneficial in improving performance under low data regimes. This situation may arise for challenging tasks where the upstream task benefits from different features. Increasing the amount of data in the upstream task did not influence the classification performance of the downstream task (**Figure 1**). A plateau effect was observed: the model ceased to learn anything new after reaching maximum accuracy for the task. Sex classification yielded better results than age prediction for the downstream task marginally. This may be because sex classification and amyloid positivity classification each involve binary classification (female/male and amyloid negative/positive, respectively) with a binary cross-entropy loss function in the final layer. In contrast, age prediction involves a regression model with a linear activation function, and the loss being minimized is the mean squared error (MSE). This model is then converted to a classification model for the downstream task, with the loss function changed to binary cross-entropy.

Another potential explanation is the difference in the ages of participants between ADNI and the UK Biobank. UK Biobank subjects are, on average, younger and may exhibit less overall atrophy on their brain MRI scans. Consequently, features learned during pre-training on age prediction may not be as beneficial in the downstream task.

7. 3D DenseNet121 Experiment and Results:

We used a 3D DenseNet121 architecture (**Figure 2**) as outlined in [40, 56, 57] for performance comparison, as it is commonly employed in the deep learning literature. The DenseNet architecture achieves excellent performance based on its connectivity pattern and feature re-use (including the use of features at multiple scales). DenseNet has multiple dense blocks and transition layers. In a dense block, each layer is connected to all prior layers, enabling feature re-use. This is achieved through concatenation, where each layer's output is concatenated with the feature maps from all prior layers. A dense block typically consists of several convolutional blocks, each producing the same number of output channels. To control model complexity and reduce the number of parameters, transition layers are introduced. These layers include convolution and pooling operations to downsample the feature maps. Model performance was evaluated using test accuracy and F1 Score. DenseNet architectures are known for their

efficiency in training and parameter sharing, making them effective for many computer vision tasks.

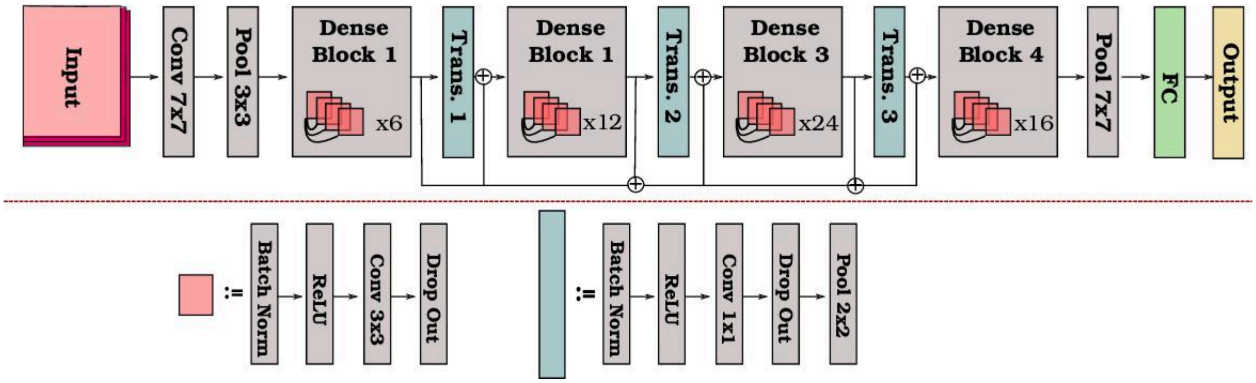

Figure 2. 3D DenseNet 121 Architecture, reproduced from [55].

The 3D DenseNet model achieved its highest accuracy of 0.773 with a test ROC-AUC of 0.826 for the image size 64x64x64.

Table 2. Experimental Results for DenseNet. The experiments are compared using test ROC-AUC, accuracy and F1 Score. **Bold numbers** indicate the best results.

Arch.	Image Size	Test ROC-AUC	Test Balanced Accuracy	Test F1 Score
DenseNet 121	$(64)^3$	0.826	0.773	0.792
	$(128)^3$	0.720	0.678	0.687
